# Whole Chromosome Instability induces senescence and promotes SASP

**DOI:** 10.1038/srep35218

**Published:** 2016-10-12

**Authors:** Grasiella Angelina Andriani, Vinnycius Pereira Almeida, Francesca Faggioli, Maurizio Mauro, Wanxia Li Tsai, Laura Santambrogio, Alexander Maslov, Massimo Gadina, Judith Campisi, Jan Vijg, Cristina Montagna

**Affiliations:** 1 Departments of Genetics, Albert Einstein College of Medicine, New York, United States; 2 Institute of Tropical Pathology and Public Health, Federal University of Goias (UFG), Goiania, GO, Brazil; 3 Translational Immunology Section, Office of Science and Technology, National Institute of Arthritis Musculoskeletal and Skin Diseases, National Institutes of Health, Bethesda, Maryland, USA; 4 Pathology, Albert Einstein College of Medicine, New York, United States; 5 Buck Institute for Research on Aging, 8001 Redwood Boulevard, Novato, California, USA; 6 Ophthalmology and Visual Science, Albert Einstein College of Medicine, New York, United States; 7 Obstetrics & Gynecology and Women’s Health, Albert Einstein College of Medicine, Yeshiva University, Bronx, NY, USA

## Abstract

Age-related accumulation of ploidy changes is associated with decreased expression of genes controlling chromosome segregation and cohesin functions. To determine the consequences of whole chromosome instability (**W-CIN**) we down-regulated the spindle assembly checkpoint component BUB1 and the mitotic cohesin SMC1A, and used four-color-interphase-FISH coupled with BrdU incorporation and analyses of senescence features to reveal the fate of W-CIN cells. We observed significant correlations between levels of not-diploid cells and senescence-associated features (**SAFs**). W-CIN induced DNA double strand breaks and elevated oxidative stress, but caused low apoptosis. SAFs of W-CIN cells were remarkably similar to those induced by replicative senescence but occurred in only 13 days versus 4 months. Cultures enriched with not-diploid cells acquired a senescence-associated secretory phenotype (SASP) characterized by IL1B, CXCL8, CCL2, TNF, CCL27 and other pro-inflammatory factors including a novel SASP component CLEC11A. These findings suggest that W-CIN triggers premature senescence, presumably to prevent the propagation of cells with an abnormal DNA content. Cells deviating from diploidy have the ability to communicate with their microenvironment by secretion of an array of signaling factors. Our results suggest that aneuploid cells that accumulate during aging in some mammalian tissues potentially contribute to age-related pathologies and inflammation through SASP secretion.

Cellular senescence is a stress response that entails an irreversible cessation of mitotic activity. As such, the senescence response is a potent tumor suppressive mechanism, but has also been implicated in the loss of physiological functions and increased disease risk associated with aging. Among the inducers of cellular senescence, particularly in human cells, is the telomere attrition that convey repeated cell division in the absence of telomerase, as well as other forms of DNA damage, most notably DNA double-strand breaks and oxidative stress (**OS**)^1^. Senescent cells also undergo widespread changes in gene expression, ultimately activating the Senescence-Associated Secretory Phenotype (**SASP**). The SASP comprises several soluble and insoluble factors that can affect the surrounding cells by activating cell-surface receptors and signal transduction pathways, which may lead to age-related pathologies, including cancer^2^.

Numerical whole chromosome instability (**W-CIN**) is a cellular state with a high propensity for chromosome mis-segregation generated by defects in the mitotic machinery and in cellular pathways controlling chromosome segregation, such as the Spindle Assembly Checkpoint (**SAC**) and sister chromatid cohesion^3^,^4^. Accordingly, deficient expression of components from either pathway results in W-CIN *in vitro* and *in vivo*^5^,^6^,^7^,^8^. Several mammalian tissues undergo changes in ploidy during normative aging, such as the brain^9^, liver^10^, lymphocytes^11^, oocytes^12^, lungs, kidney and heart^13^. Interestingly, decreased levels of SAC, cohesin and kinetochore proteins are observed in tissues that undergo age-related aneuploidization^3^,^14^,^15^, which could be causative of W-CIN generation at older age.

Evidence for W-CIN activating the senescence response has emerged from studies suggesting that down-regulating genes that result in aneuploidy induces premature senescence in human cells^6^,^7^,^16^,^17^. Aneuploidy resulting from lagging chromosomes^13^, or from a polyploid intermediate state^18^,^19^, has been associated with senescence and premature aging in mice, reinforcing the potential role of W-CIN in age-related tissue degeneration. Yet, a clear causal role of how W-CIN could drive aging phenotypes has not been established.

To directly test if W-CIN is sufficient to trigger cellular senescence, we down-regulated the SAC component BUB1 mitotic checkpoint serine/threonine kinase (*BUB1*) and the mitotic cohesin component structural maintenance of chromosomes 1A (*SMC1A)* in human primary fibroblasts (**HPF**) and evaluated single cell ploidy by a custom designed four-color-interphase Fluorescence *In Situ* Hybridization (**FISH**) approach previously described by us^9^,^20^. We observed significant correlation between levels of not-diploid (**Not 2n**) cells and senescence-associated features (**SAFs**). Moreover, W-CIN induced DNA double strand breaks (DSBs), OS and a SASP that comprises factors associated with senescence induction through both DNA damage and mitochondrial dysfunction. In addition, W-CIN induced the secretion of a growth factor, C-type lectin domain family 11 member A (CLEC11A/SCGF-b), which has not been previously associated with SASP and its secretion level is correlated with the frequency of not 2n cells. The present study proposes a model in which W-CIN triggers senescence arrest by multiple non-exclusive pathways, and suggests, for the first time, that cells deviating from diploidy can affect their microenvironment *via* secretion of SASP.

## Results

### Replicative senescence (SEN) of cultured HPF is coupled with reduction in expression levels of SAC components *BUB1, BUB1B, BUB3* and cohesin *SMC1A*

**Figure 1:**
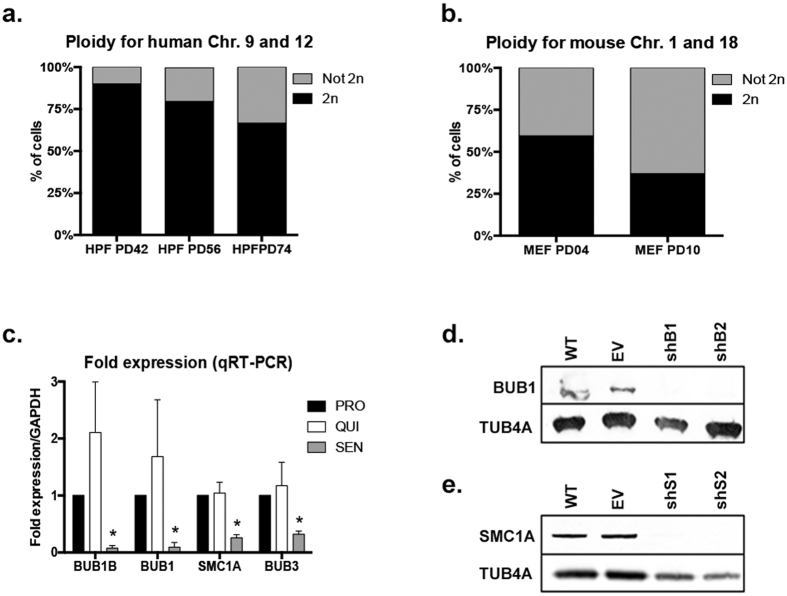
Mammalian cells undergo ploidy changes and down-regulation of SAC and cohesin components as they approach SEN in culture. (**a**) Two-chromosomes FISH analysis of ploidy of human primary fibroblasts (HPF) was performed at early (PD42), mid (PD56) and late PDs/SEN (PD74). Plots depict the percentage of cells that were found diploid (2n, black) or not-diploid (Not 2n, gray). (**b**) Two-chromosomes FISH analysis of ploidy performed on mouse embryonic fibroblasts (MEFs) at early (PD04) and late PDs/SEN (PD10). (**c**) Gene expression analysis depicting mRNA levels of *BUB1B, BUB1, SMC1A* and *BUB3* in non-mitotic quiescent cells (QUI-white) and SEN cells (SEN-grey) relative to proliferating cells (PRO-black). Down-regulation of the tested genes is statistically significant only in SEN cells. (**d,e**) Lentiviral delivery of shRNAs results in knockdown of *BUB1* and *SMC1A* in IMR-90 fibroblasts as detected by western blotting. (**d**) Knockdown of *BUB1* and (**e**) *SMC1A* at the protein level (EV = Empty vector infected cells). TUB4A was used as loading control. (*) Indicates significant differences (*p* < 0.05) from PRO cells tested by One-way ANOVA. Data are expressed as mean ± SD (n = 3).

Primary mammalian cells *in vitro* show accumulation of ploidy changes as they approach SEN (Fig. 1a,b, Supplementary Table S1 and Figure S1). Therefore, we investigated if down-regulation of four major components of the chromosome segregation machinery (BUB1B, BUB1, SMC1A and BUB3) occurs during SEN of HPF *in vitro*. We found that all mitotic components tested were significantly down-regulated at the mRNA level (*p* = 0.0219 for BUB1B*, p* = 0.0405 for BUB1*, p* = 0.0333 for SMC1A and *p* = 0.0357 for BUB3) in SEN cells relative to proliferating cells (Fig. 1c). Moreover, decrease in gene expression appeared SEN-specific, as non-mitotic quiescent cells did not significantly deregulate the expression of those targets (*p* = 0.3294 for BUB1B*, p* = 0.5093 for BUB1*, p* = 0.7947 for SMC1A and *p* = 0.6539 for BUB3) (Fig. 1c). Both *BUB1B* and *BUB1* were down-regulated more than 10 fold in SEN when compared to proliferating cells (13 fold and 10 fold, respectively), while *SMC1A* and *BUB3* were down-regulated to a lesser extent (3.9 and 3.1 fold, respectively). These results are in agreement with SAC and cohesin proteins being expressed at lower levels at older age^3^,^7^,^14^,^15^, and suggest that the senescence-associated accumulation of ploidy changes in cultured fibroblasts could be a consequence of limited availability of those components.

### *BUB1* and *SMC1A* depletion result in decreased proliferation and W-CIN generation

In order to test the hypothesis that changes in ploidy are sufficient to trigger the senescence response, we established an *in vitro* system to induce W-CIN by dampening the expression of genes that we found down-regulated during SEN. We selected *BUB1* and *SMC1A* because of their suggested involvement in age-related ploidy changes^3^,^14^,^15^ and because they perform distinct functions to prevent chromosome mis-segregation^21^,^22^. For knockdown studies in HPF (IMR-90 cells) we used a lentiviral delivery system to express shRNAs targeting these components. Cells were transduced at early passage (~35 population doublings - PDs) to ensure maximum proliferative capabilities and to avoid the presence of ploidy changes due to pre-senescence confounding factors. As a negative control a vector without shRNA (empty vector - EV) was delivered to early passage cells, and as positive control we used replicative senescent cells (SEN – PD74.5 SD ± 0.6). To control for off-target effects, two shRNAs were used for each candidate gene (herein referred to as shB1 and shB2 targeting *BUB1*, and shS1 and shS2 against *SMC1A*). Cells were harvested 13 days after lentiviral transduction, and gene knockdown was assessed by Western Blot to confirm efficient down-regulation of both BUB1 and SMC1A (Fig. 1d,e).

**Figure 2:**
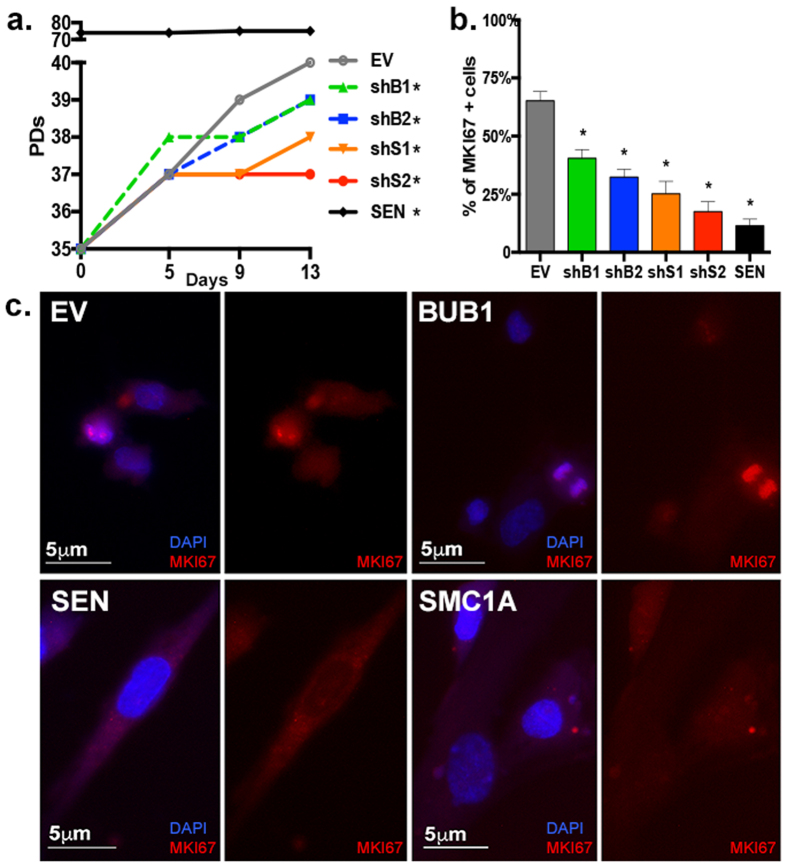
Proliferation of HPF is impaired upon *BUB1* or *SMC1A* knockdown. (**a**) Growth curve of EV, BUB1 or SMC1-depleted and SEN cultures as assessed by number of PDs. (**b**) Quantification of percentage of MKI67 positively stained cells per cell line. (**c**) Representative IF images for the proliferation marker MKI67 (red) in EV, SEN, BUB1 and SMC1A-depleted cells. (*) Indicates significant differences (*p* < 0.05) from EV cells tested by One-way ANOVA. Data are expressed as mean ± SD (n = 3).

To begin functional characterization, the growth curve of each cell line was determined over the course of 13 days by calculating the respective PDs every 3–4 days. As expected, SEN cultures did not increase PDs during this time (Fig. 2a). Both BUB1 (shB1: PD39.2 SD ± 0.9 and shB2: PD38.7, SD ± 0.7) and SMC1A-depleted cells (shS1: PD.37.5, SD ± 0.3 and shS2: PD36.8, SD ± 0.2) exhibited slower proliferation relative to EV (PD40.5 SD ± 1), with the latter presenting a more pronounced effect (Fig. 2a). Nonetheless, all depleted cultures reached significantly lower PDs relative to EV (shB1: *p* = 0.0386; shB2: *p* = 0.0055; shS1: *p* = 0.0002; shS2 and SEN: *p* < 0.0001) (Fig. 2a). We next performed immunofluorescence (IF) analyses for the marker of proliferation Ki-67 (MKI67) (Fig. 2b,c). We observed significantly reduced amounts of MKI67-stained cells in BUB1 down-regulated cultures (~32–41%) compared to EV (65%), and even lower amounts in cultures depleted of SMC1A (~17–25%) confirming a more pronounced phenotype under this condition (*p* < 0.0001 for all cultures) (Fig. 2b). The levels of expression of MKI67 reflect the differences observed in the growth curve analysis and they indicate decreased cellular proliferation upon BUB1 or SMC1A knockdown.

**Figure 3:**
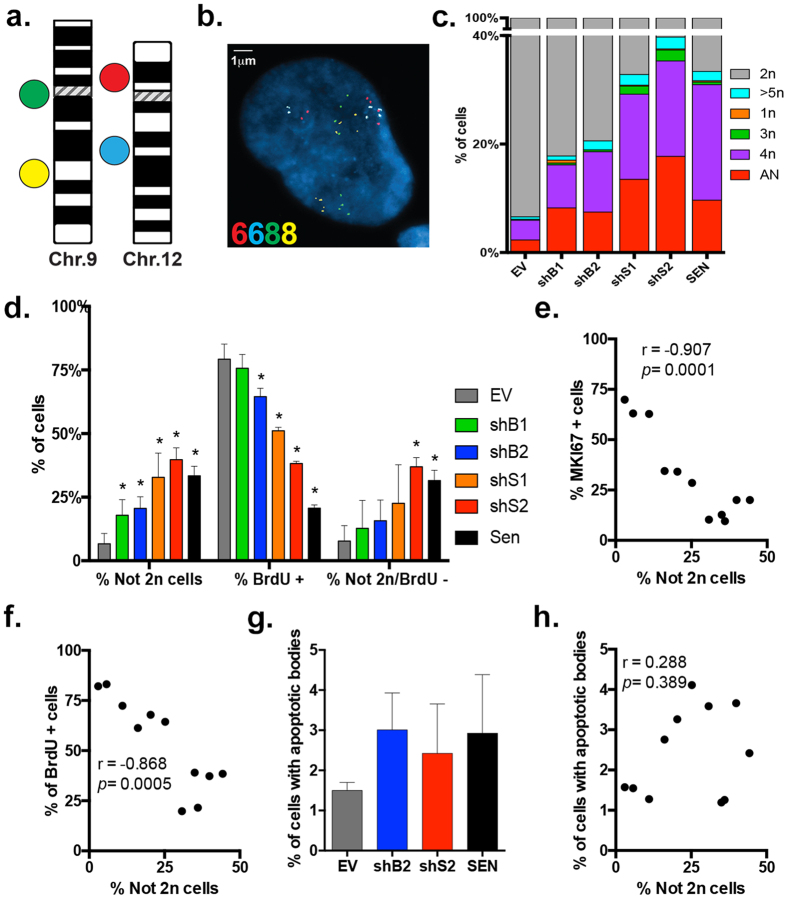
Depletion of *BUB1* or *SMC1A* results in W-CIN, decrease of DNA replication and negligible apoptosis in HPF. (**a**) Ideograms of human chromosome 9 and 12 depicting the four BAC clones and their differently labeled probes used for ploidy analysis. (**b**) Representative interphase FISH image of an aneuploid nucleus. The numbers of signals for each chromosome are indicated (bottom left) with the correspondent color of fluorophore used for staining. (**c**) Representative plotting of the distribution of cells deviating from diploidy in each sample. Aneuploid cells (AN, red) comprise nuclei with discordant numbers of signals for chromosomes 9 and 12 irrespective of their number. (**d**) Quantification of the percentage of not 2n, BrdU positively stained and not 2n/BrdU negative cells in each sample, respectively. (**e,f**) Significant correlation plots between the percentages of not 2n and (**e**) MKI67 or (**f**) BrdU positively stained cells. (**g**) Quantification of nuclei with apoptotic bodies in each cell line. (**h**) Non-significant correlation plot between the percentages of not 2n and apoptotic cells. (*) Indicates significant differences (*p* < 0.05) from EV cells tested by One-way ANOVA. Data are expressed as mean ± SD (n = 3).

Knockdown of *BUB1* or *SMC1A* generates W-CIN^5^,^7^, we therefore quantified the level of ploidy changes in human fibroblasts after 13 days of stable down-regulation. Because depletion of *BUB1* and *SMC1A* was expected to trigger cell cycle arrest, we bypassed analysis of metaphases that could potentially skew the results and performed, instead, a custom designed four-color interphase Fluorescence *In Situ* Hybridization (FISH) approach^9^,^20^. The combined use of two pairs of probes specific for chromosome 9 (yellow and green signals in Fig. 3a) and chromosome 12 (blue and red signals in Fig. 3a) at two separate loci allows a sensitive and highly quantitative analysis of chromosome numerical changes, as well as identification of aneuploidy versus polyploidy. Approximately 200 nuclei per culture in three biological replicates were analyzed. Nuclei were scored as diploid when two copies of each chromosome specific locus signals were observed (Supplementary Fig. S2a), or polyploid when containing multiples of the haploid complement (Supplementary Fig. S2b,c). Nuclei were scored as aneuploid when the number of signals for chromosome 9 was discordant from those of chromosome 12 (Fig. 3b). Nuclei were also scored for ploidy relative to 24 h BrdU incorporation in order to determine if cells that deviate from diploidy also halt DNA synthesis (Supplementary Fig. S2d,e).

EV cultures were mainly diploid (93.4% SD ± 4.1) and presented a high frequency of cells positive for BrdU incorporation (79.2% SD ± 5.9), while SEN cultures had significantly decreased percentages of diploid (66.6% SD ± 3.7, *p* = 0.0004) and BrdU incorporating cells (20.7% SD ± 1.3, *p* < 0.0001) (Fig. 3c,d). The substantial amount of SEN cells that are BrdU positive (20.7%) is likely due to their arrest in both G1 and G2 phases of the cell cycle^23^, and is in agreement with observations of residual DNA synthesis in SEN cultures despite little or no increase in cell number^24^. In general, fibroblasts depleted of *SMC1A* were less diploid and presented fewer replicating cells than the ones depleted of *BUB1*: shS1 and shS2 contained 67.2% and 60.3% (SD ± 9.5, 4.6) of diploid cells and 51.1% and 38.3% (SD ± 1.4, 0.9) of BrdU incorporating cells, respectively. shB1 and shB2 contained 82.1% and 79.4% of diploid cells (SD ± 6.1, 4.6) and 75.7% and 64.7% (SD ± 5.4, 3.5) of BrdU incorporating cells, respectively (see Supplementary Table S2). All cultures depleted of either gene had significantly higher amounts of not 2n cells than EV (shB1: *p* = 0.0397; shB2: *p* = 0.0148; shS1: *p* = 0.0002; shS2: *p* < 0.0001), confirming findings by others^5^,^7^ (Fig. 3c,d). The not 2n population generated by the knockdown of either target comprised aneuploid, triploid and polyploid cells (see Supplementary Table S2). Moreover, nuclei harboring only 1 copy of the chromosomes tested were rare (0.5–1.6%), suggesting that monosomy is poorly tolerated.

We also investigated if there was a relationship between arrest of DNA replication and deviation from diploidy by analyzing the percentage of cells lacking BrdU staining that were not 2n in each sample. Cultures that mostly deviate from diploidy (shS2 and SEN) were statistically significant for the enrichment of not 2n cells in the BrdU negative population (shS2: *p* = 0.0027; SEN: *p* = 0.0174) (Fig. 3d). Correlation analysis between ploidy and proliferation, as assessed by MKI67 and BrdU incorporation staining, have negative and significant Pearson correlation coefficients (r) (r = −0.907, r = −0.868; *p* = 0.0001, *p* = 0.0005, respectively), indicating that there is a trend for reduced proliferation with increased levels of W-CIN (Fig. 3e,f and Supplementary Table S3). These results suggest that human cells depleted from *BUB1* or *SMC1A* are prone to ploidy changes that are accompanied by a reduction in proliferation and DNA replication.

Next, we performed statistical t-test (*p* < 0.05; two-tailed) between the two cell lines down-regulated for each gene (BUB1: shB1 vs. shB2 and SMC1A: shS1 vs. shS2) to verify if their proliferation status (assessed by MKI67 expression) or their W-CIN levels (assessed by FISH) were discordant. Because there were no significant differences in the percentage of cells expressing MKI67 at 13 days (shB1 vs. shB2: *p* = 0.0613 and shS1 vs. shS2: *p* = 0.1728) or in W-CIN levels (shB1 vs. shB2: *p* = 0.661 and shS1 vs. shS2 *p* = 0.1336) measured between the 2 different shRNAs targeting the same gene, we proceeded with further analyses using shB2 and shS2 due to their higher deviation from diploidy.

### Apoptosis is a minor outcome of W-CIN induced by *BUB1* or *SMC1A* depletion

The proliferation of aneuploid cells is constrained by a TP53-dependent mechanism^25^,^26^, which induces either apoptosis^5^,^20^,^25^ or cell cycle arrest and senescence^6^,^16^,^17^. Apoptosis is morphologically characterized by the presence of apoptotic bodies that appears as visible nuclear fragments^27^. To verify if cell death was one of the outcomes of *BUB1* or *SMC1A* depletion, we analyzed nuclei for the presence of apoptotic bodies (see Supplementary Fig. S2f). The occurrence of fragmented nuclei was overall low, with 1.1% observed in EV cultures (SD ± 0.7) and 2.9%, 3%, and 2.4% (SD ± 1.5, 0.9, 1.2) observed in SEN, shB2, and shS2 cultures, respectively (Fig. 3g). The slight increase in levels of cell death upon knockdown of BUB1 or SMC1A was not significantly different from EV cultures (shB2: *p* = 0.093; shS2: *p* = 0.3094; SEN: *p* = 0.1306) and remained below 4%. Moreover, correlation analysis between ploidy and occurrence of apoptotic bodies was not significant (r = 0.288, *p* = 0.389) (Fig. 3h and Supplementary Table S3), suggesting that apoptosis is not the main outcome of W-CIN in this system.

### *BUB1* or *SMC1A* knockdown increase the expression of SAFs

**Figure 4:**
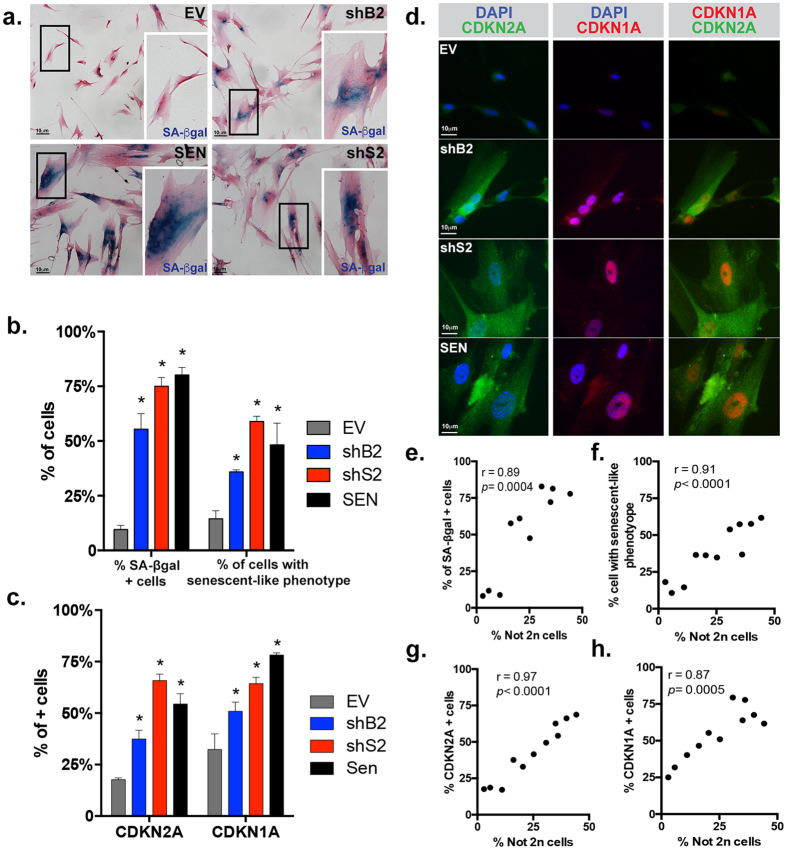
*BUB1* or *SMC1A* knockdown increase the expression of Senescence-Associated Features (SAFs). (**a**) Bright field representative images of IMR-90 fibroblasts stained for SA-βgal and counterstained with eosin. Zoom-in of highlighted islets is shown on the bottom right corner. (**b**) Quantification of the percentage of cells positively stained for SA-βgal and with a senescent-like phenotype assessed by FACS, respectively. (**c**) Quantification of the percentage of cells positively stained for CDKN2A and CDKN1A, respectively. (**d**) Representative images of double IF for CDKN2A (green) and CDKN1A (red). (**e–h**) Significant correlation plots between the percentages of not 2n and (**e**) SA-βgal, (**f**) senescent-like phenotype, (**g**) CDKN2A or (**h**) CDKN1A positively stained cells. (*) Indicates significant differences (*p* < 0.05) from EV cells tested by One-way ANOVA. Data are expressed as mean ± SD (n = 3).

To further investigate the fate of *BUB1* or *SMC1A*-depleted fibroblasts, we performed Senescence-associated β-galactosidase (SA-βgal) staining to identify senescent cells (Fig. 4a)^28^. We found that knockdown of either genes resulted in significant increase of SA-βgal positive cells (*p* < 0.0001 for all cultures) when compared to EV (EV = 9.6% SD ± 1.9, shB2 = 55.4 SD ± 7.1, shS2 = 74.7% SD ± 2.86, SEN = 80.2% SD ± 3.42) (Fig. 4b). Cells depleted of either gene also underwent drastic changes in morphology reminiscent of SEN, such as larger size and flatter morphology (islets in Fig. 4a). Another characteristic of SEN cells is the accumulation of lipofuscin, a highly oxidized conglomerate of cross-linked proteins, lipids, sugars and metals, which results in increased cellular autofluorescence (AF)^29^. When analyzed by FACS for a senescent-like phenotype (i.e. elevated size and/or AF) (Supplementary Fig. S3)^30^, we observed significantly higher number of cells bearing senescent-like features in BUB1 and SMC1A-depleted cultures relative to EV (shB2: *p* = 0.0013; shS2: *p* < 0.0001), similar to what observed in SEN cultures (SEN: *p* < 0.0001) (Fig. 4b).

Two tumor suppressor proteins, cyclin-dependent kinase inhibitor 2A (CDKN2A) and cyclin-dependent kinase inhibitor 1A (CDKN1A), are known to mediate the senescence cell cycle arrest^1^. We therefore assessed their expression by IF and determined that 54.4% and 78.2% (SD ± 5.0, 1.0) of SEN cells and 17.8% and 32.3% (SD ± 0.8, 7.6) of EV cells stained positive for CDKN2A and CDKN1A, respectively. Cultures depleted of BUB1 and SMC1A were significantly enriched for the frequency of CDKN2A (37.4% SD ± 4.3, *p* = 0.002 and 65.9% SD ± 3.0, *p* < 0.0001, respectively) and for CDKN1A (50.9% SD ± 4.4, *p* = 0.0012 and 64.3% SD ± 3.0, *p* < 0.0001, respectively) positive cells (Fig. 4c,d). These results suggest the involvement of both the CDKN2A/RB1 and the TP53-CDKN1A pathways in the senescence-like growth arrest upon induction of W-CIN.

Correlation analyses between ploidy and SAFs have positive and significant r values (%SA-βgal: r = 0.89, *p* = 0.0004; %Senescent-like: r = 0.91, *p* < 0.0001; %CDKN2A: r = 0.97, *p* < 0.0001; %CDKN1A: r = 0.87, *p* = 0.0005), indicating increased expression of senescent markers as W-CIN levels increase (Fig. 4e–h and Supplementary Table S3).

### W-CIN-associated DNA damage and OS likely contribute to senescence induction

**Figure 5:**
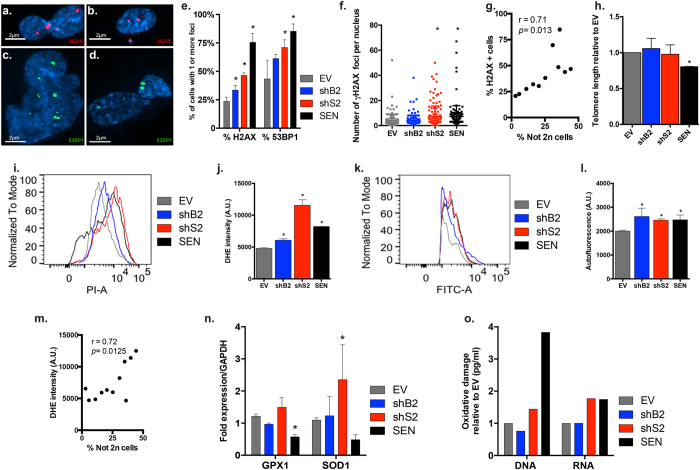
W-CIN-induced senescence is associated with DNA DSBs generation and OS. (**a–d**) Representative images of IF of γH2AX (red) and 53BP1 (green) foci in W-CIN cells depicting (**a**) a nucleus with chromatin bridging, (**b**) a micronucleus extruded from main nucleus, (**c**) an abnormally shaped nucleus and (**d**) a binucleated cell. (**e**) Quantification of the percentage of cells containing 1 or more γH2AX and 53BP1 foci, respectively. (**f**) Quantification of the number of γH2AX foci per nucleus in each sample. (**g**) Correlation plot between the percentages of not 2n and γH2AX foci containing cells. (**h**) Telomere length relative to EV cells measured by qPCR. (**i**) Representative histogram of DHE intensity (PI-A channel) showing all samples identified by their respective colors (EV: gray, shB2: blue, shS2: red, SEN: black). (**j**) Quantification of the mean DHE intensities. (**k**) Representative histogram of AF intensity (FITC-A channel) showing all samples identified by their respective colors (as above). (**l**) Quantification of the mean AF intensities. (**m**) Correlation plot between the percentages of not 2n and DHE intensity. (**n**) Fold expression of antioxidant enzymes GPX1 and SOD1 mRNA levels, normalized to GAPDH. (**o**) Fold differences of oxidative damage measured in DNA and RNA relative to EV. (*) Indicates significant differences (*p* < 0.05) from EV cells tested by One-way ANOVA. Data are expressed as mean ± SD (n = 3).

Next, we sought to investigate if known senescence stimuli (i.e. DNA DSBs, telomere shortening and OS)^1^ were involved in W-CIN-induced senescence. A persistent DNA damage response (DDR) has been shown to initiate senescence resulting from unrepairable DSBs at critically eroded telomeres or at non-telomeric sites^1^. Among the proteins that assemble at DNA lesions to facilitate checkpoint activation and repair are the H2A histone family member X (H2AX) and the p53 binding protein 1 (53BP1), which form stable focal aggregates and are commonly used to indicate persistent DNA damage^31^. Because abnormal mitosis and W-CIN are associated with the acquisition of DNA damage^3^,^32^,^33^, we performed IF analysis of γH2AX and 53BP1 foci to measure levels of W-CIN-induced genomic damage in cells depleted of BUB1 or SMC1A (Fig. 5a–d). We observed higher amounts of cells containing at least 1 focus formation of each marker in both shB2 (~33% and 61%, respectively) and shS2 cultures (46% and 71%, respectively) when compared to EV (~23% and 43%, respectively) (Fig. 5e), however shB2 was only significant for the percentage of γH2AX foci positive cells (*p* = 0.042). Likewise, only shS2 and SEN cultures carried significantly more foci per nucleus than EV cells (γH2AX: *p* < 0.0001 for both; 53BP1: *p* = 0.0172 for shS2 and *p* < 0.0001 for SEN) (Fig. 5f and Supplementary Fig. S4a). Correlation analyses between ploidy and DNA DSBs have positive and significant r values (% γH2AX: r = 0.71, *p* = 0.013 and %53BP1: r = 0.73, *p* = 0.01) (Fig. 5g, Supplementary Table S3 and Fig. S4b), suggesting higher incidence of DSBs as W-CIN levels increases. The results imply that W-CIN generated upon knockdown of BUB1 and SMC1A can indirectly produce DSBs that in turn, may elicit the senescence arrest.

Progressive shortening of telomeres through continuous cell division ultimately leads to irreversible cell cycle arrest of primary human cells^1^. To rule out the possibility of telomere erosion in our system, we measured telomere length in all cultures by qRT-PCR^34^. The telomeres of SEN cells, as expected, were significantly shorter than EV cells (*p* = 0.0082) (Fig. 5h). However, the telomeres of cells depleted of *BUB1* or *SMC1A* for 13 days were not significantly more eroded than those measured in EV cells (shB2: *p* = 0.6753; shS2: *p* = 0.849) (Fig. 5h). Telomere shortening was, therefore, not a cause of premature senescence in this system.

W-CIN is associated with a state of elevated OS^35^ and cells deviating from diploidy undergo metabolic alterations^36^ and increased production of Reactive Oxygen Species (**ROS**)^37^,^38^ that can cause oxidative genomic damage^25^,^39^. We, therefore, investigated if OS was associated with senescence induction after BUB1 or SMC1A down-regulation. We measured ROS production by staining the cells with the Dihydroethidium (DHE) dye and measuring the respective mean intensities by FACS (PI-A channel)^40^ (Fig. 5i). Both BUB1 and SMC1A-depleted cultures exhibited significantly higher DHE intensity when compared to EV (shB2: *p* = 0.0343; shS2: *p* < 0.0001), in addition shS2 cells produced even higher superoxide levels than SEN cells (Fig. 5j). Correlation analysis between ploidy and DHE intensity have positive and significant r value (r = 0.72, *p* = 0.0125) (Fig. 5m and Supplementary Table S3), suggesting that ROS production increase with W-CIN levels. Elevated OS can accelerate lipofuscin build up^29^; therefore we also analyzed the intensity of AF by FACS (FITC-A channel) (Fig. 5k). We observed significantly accumulation of lipofuscin relative to EV in all cultures (shB2: *p* = 0.0061; shS2: *p* = 0.0246; SEN: *p* = 0.0216) (Fig. 5l). Because high amounts of free radicals can be detrimental to cellular components, antioxidant defense mechanisms evolved to maintain redox homeostasis^41^. Cells generally respond to excess ROS by up-regulating antioxidant enzymes that neutralize them, such as Glutathione Peroxidase (GPX) and Superoxide Dismutase (SOD)^38^. We, therefore, analyzed if the mRNA levels of the antioxidant enzymes GPX1 and SOD1 were up-regulated as a consequence of W-CIN. Only shS2 cells expressed higher levels of both enzymes than EV, SOD1 being significant (*p* = 0.039) (Fig. 5n). Conversely, SEN cells were down-regulated for both, in agreement with a decreased ability to induce transcription of antioxidant enzymes during aging, as previously shown in aged oocytes^42^. Cells deviating from diploidy accumulate oxidative DNA damage as a consequence of increased ROS levels^25^,^39^. Thus we verified if BUB1 or SMC1A down-regulated cells contained oxidized nucleic acids by detecting three oxidized guanine species: 8-hydroxy-2′-deoxyguanosine (8-oxo-dG) from DNA, 8-hydroxyguanosine from RNA and 8-hydroxyguanine (8-oxo-Gua) from either molecules. SEN fibroblasts contained higher levels of oxidation in both their molecules relative to EV (3.8 fold for DNA and 1.74 fold for RNA) (Fig. 5o), in agreement with oxidative lesions being accumulated during aging in those^43^. Although shB2 cells had oxidation levels similar to EV, shS2 cells exhibited elevated levels of oxidized nucleic acids relative to EV (1.4 fold for DNA and 1.77 fold for RNA). Strikingly, their RNA oxidation levels were similar to SEN cells, even though shS2 cells were cultured for only 13 days (a time period 9 fold shorter than SEN cells). This is likely due to the fact that RNA is more vulnerable to oxidative damage than DNA^44^. Taken together, these results reinforce the hypothesis that W-CIN is associated with a state of OS which likely affects the transcriptome more severely, ultimately representing a signal triggering cell cycle arrest, as previously suggested^25^,^37^,^39^.

### W-CIN-induced senescence promotes the secretion of SASP factors

The irreversible cell cycle arrest triggered by DNA damage, eroded telomeres or oncogene activation is accompanied by a SASP – the secretion of soluble signaling factors (interleukins, chemokines and growth factors), proteases and insoluble extracellular matrix components that can affect senescent cells and their microenvironment by activating signal transduction pathways^1^,^2^. To determine whether W-CIN-induced senescence also activates a SASP, we initially focused the analysis on shS2 cultures, which showed the highest deviation from diploidy and strongest senescent phenotypes. In a preliminary screen, we analyzed the conditioned medium (CM) of EV and shS2 cultures for the levels of 48 cytokines, chemokines and growth factors and found that 17 of those tested were secreted 2 fold or more in shS2 cells (for full gene names, refer to Supplementary Table S4). Of these, 16 were previously identified as SASP components^2^,^45^,^46^,^47^. A novel growth factor, CLEC11A/SCGF-b, which to our knowledge has not yet been associated with senescence yet, showed a trend for increased secretion in shS2 cells. Of interest, W-CIN-induced SASP comprised factors signatures of both DNA damage (CXCL8, CSF2, CCL2, IL6)^48^,^49^ and mitochondrial dysfunction-induced senescence (TNF, CCL27 and IL10)^47^. In this initial screening 4 factors reached statistical significance (*p* < 0.05): CCL2, IL1B, TNF and CSF2 (see Supplementary Table S4).

**Figure 6:**
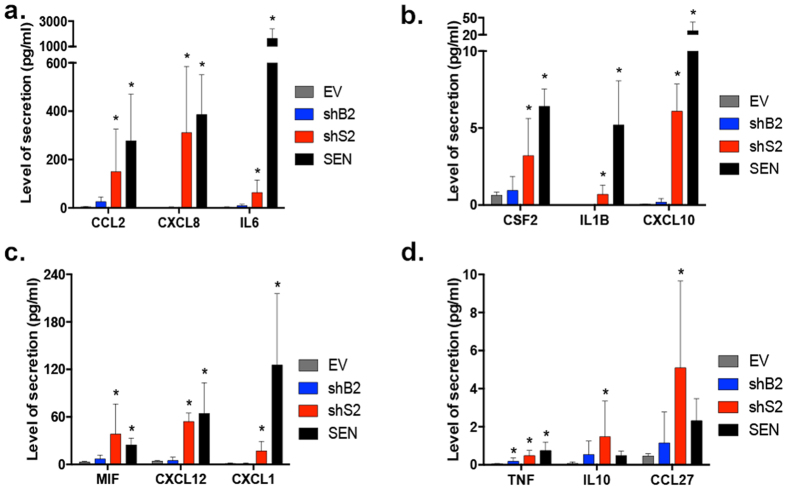
W-CIN promotes secretion of SASP factors comprising signatures of DNA damage and mitochondrial dysfunction-induced senescence. Validated SASP components secreted in the conditioned medium (CM) of EV, shB2, shS2 and SEN cells. (**a–c**) Characteristic DNA damage-induced senescence SASP factors measured in CM: (**a**) CCL2, CXCL8, IL6; (**b**) CSF2, IL1B, CXCL10; (**c**) MIF, CXCL12, CXCL1. SASP factors signature of mitochondrial dysfunction-induced senescence: (**d**) TNF, IL10, CCL27. (*) Indicates significant differences (*p* < 0.05) from EV cells tested by One-way ANOVA. Data are expressed as mean ± SD (n = 6, n = 5 for SEN).

**Figure 7:**
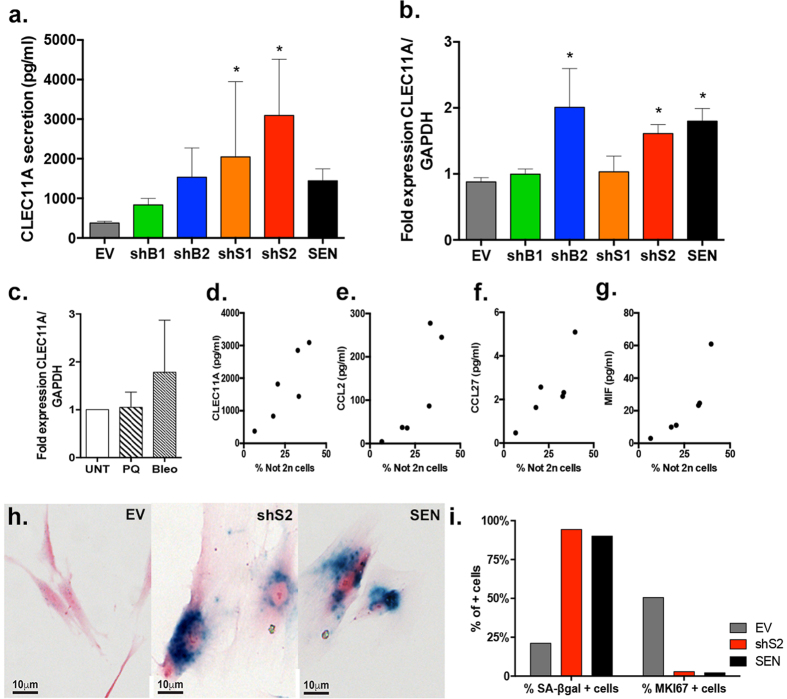
W-CIN induced senescent cells secrete CLEC11A. (**a**) Levels of CLEC11A secreted in the CM of each SMC1 and BUB1-depleted cell line. (**b**) Fold expression of CLEC11A mRNA levels normalized to GAPDH in W-CIN-induced senescent and SEN cells and (**c**) Paraquat (PQ, black and white stripe bar) or Bleomycin (Bleo, gray bar)-induced senescence relative to untreated cells (UNT, white bar). (**d–g**) Secretion levels of SASP factors that significantly correlate with W-CIN levels: (**d**) CLEC11A, (**e**) CCL2, (**f**) CCL27 and (**g**) MIF. (**h**) Bright field representative images of SA-βgal staining in EV, shS2 and SEN fibroblasts 45 days after lentiviral transduction. (**i**) Quantification of SA-βgal and MKI67 positively stained fibroblasts 45 days after lentiviral transduction. (*) Indicates significant differences (*p* < 0.05) from EV cells tested by One-way ANOVA. Data are expressed as mean ± SD (n = 3).

Based on the statistically significant SASP components found in shS2 cultures or secreted proteins that had the highest fold difference relative to the EV, we generated a new custom array for testing the secretion of 13 factors in additional CM replicates obtained from all cell lines used in our study (see Supplementary Table S5). As expected, and validating our previous screening, shS2 and SEN cells up-regulated all these 13 factors albeit at various levels (Supplementary Figure S5). Based on one-way ANOVA analysis comparing all samples to EV, all 13 components were significantly more secreted by shS2 cells (*p* < 0.05) (Fig. 6 and Supplementary Figure 5), including CLEC11A (*p* = 0.0042) (Fig. 7a). Significant secretion of TNF occurred in shB2 cultures (*p* = 0.201), while CXCL10 (*p* = 0.0079) and MIF (*p* = 0.0499) were also secreted by shS1 cells (Supplementary Figure S5). Some SASP factors also showed a trend for up-regulation in the other shRNA cell lines (CCL2, CCL27, CSF2, CXCL12, IL10) however not significant compared to EV (Supplementary Table S5 and Figure S5).

In addition to shS2 cells, the novel identified SASP factor CLEC11A, was also significantly more secreted by shS1 cells (*p* = 0.0132) (Fig. 7a). Analysis of differential CLEC11A secretion between groups using ANOVA did not reach statistical significance for shB1 and shB2. However, when the differences between EV and BUB1-depleted cells were analyzed using a t-test, both cell lines showed significant increased secretion of CLEC11A relative to EV (shB1: *p* = 0.0055; shB2: *p* = 0.045), suggesting that the secretion of this growth factor is a bona fide W-CIN SASP component. The up-regulation of CLEC11A was also confirmed at the mRNA level for shB2 and shS2 (Fig. 7b). The lack of association between CLEC11A and SASP in the literature, together with our findings, suggests that this factor may be specific of senescence induction through W-CIN generation. To test this hypothesis, we induced premature senescence in HPF by using two established independent approaches^31^,^50^: by causing OS through exposure to Paraquat (PQ – a superoxide inducer) and by generating DNA damage through Bleomycin treatment (Bleo – DSBs inducer). Senescent phenotype under these conditions was confirmed trough SA-βgal staining (Supplementary Figure S6), and CLEC11A mRNA levels were measured using qPCR. Neither PQ nor Bleo-induced senescence caused significant up-regulation of CLEC11A relative to untreated (UNT) cells (PQ: *p* = 0.9284; Bleo: *p* = 0.1941) (Fig. 7c) supporting our finding that this factor may be specific of W-CIN induced senescence. Moreover, correlation analysis between the levels of CLEC11A and the levels of not 2n cells is statistically significant (r = 0.8619, *p* = 0.0273) (Fig. 7d and Supplementary Table S6). Interestingly, this is also the case for CCL2, CCL27 and MIF (Fig. 7e–g and Supplementary Table S6), suggesting that the secretion level of these components increases along with levels of not 2n cells. Taken together, these results imply that cells deviating from diploidy acquire the ability to communicate with their microenvironment and affect neighboring cells through the secretion of soluble cytokines, chemokines and growth factors, likely through the process of senescence. In addition, our data uncover a new SASP component, CLEC11A, which we propose as being induced by W-CIN.

### W-CIN-induced senescent fibroblasts persist in culture long-term after depletion of *SMC1A*

All cultures analyzed in this study were heterogeneous in terms of proliferation containing varying amounts of BrdU incorporating and MKI67-expressing cells. We speculated that proliferating fibroblasts present in W-CIN-induced senescent cultures would continue dividing and eventually overgrow their senescent counterparts. Because shS2 cultures had the highest levels of not 2n cells, SAFs and SASP, we maintained these cells up to 45 days after lentiviral transduction and analyzed them for SA-βgal (Fig. 7h) and MKI67 staining. Relative to fibroblasts growing for 13 days, 45 days cultures showed increased frequency of SA-βgal activity and decreased amounts of MKI67 stained cells, consistent with a progressive passaging of primary fibroblasts *in vitro*. shS2 cultures contained an extremely high percentage of SA-βgal positive cells (94.3%), which is comparable to SEN (90%), while only 17.7% of EV cells stained positive (Fig. 7i). Accordingly, we found that only ~2–3% of shS2 and SEN cells expressed MKI67 while 50.5% of EV cells did (Fig. 7i). The similarity between shS2 and SEN cultures is striking, despite shS2 cells being in culture for only 45 days (2 fold shorter than SEN cells that have been cultured for 4 about months). These results suggest that W-CIN-induced senescent cells persist in culture and do not resume proliferation or undergo cell death, and, moreover that the non-senescent cells in the culture continue to enter senescence for up to 45 days after inducing W-CIN.

## Discussion

The purpose of this study was to test the hypothesis that W-CIN is sufficient to trigger the senescence arrest and promote SASP, in order to avoid the proliferation of cells with genomes deviating from diploidy. Previous reports suggest that aneuploidy is associated with cell cycle arrest and premature senescence^6^,^7^,^16^,^17^,^37^. However, most of the previous studies analyzed metaphase chromosome spreads^6^,^7^,^16^,^17^ which require actively proliferating cells and therefore provides inaccurate conclusions when studying senescent cells. Because senescence is a stage of irreversible cell cycle arrest, it could be argued that the high aneuploidy levels previously reported only reflected the levels of the subpopulation that is still able to engage cell division, confounding a causal role for aneuploidy as a trigger of senescence. In this study instead, we performed detailed FISH analysis on interphase nuclei allowing a more accurate quantification of W-CIN of the whole population, including permanently arrested senescent cells. We used a custom designed four-color FISH approach previously described by us, that is based on the enumeration of two separate loci on a given chromosome and it is highly sensitive and reproducible^9^,^20^.

To investigate the consequences of W-CIN we performed gene knockdown of the SAC component BUB1 and cohesin SMC1A based on the observation that they are significantly less expressed in SEN cells (Fig. 1c) and also reported down-regulated during normative aging^3^,^14^,^15^. The SAC is an important surveillance mechanism that monitors proper bipolar attachment of all kinetochores to microtubules before anaphase onset^3^. Because BUB1 is required for the localization of other checkpoints proteins, for proper chromosome congression and to maintain centromeric cohesion^22^,^51^, aneuploidy is a common outcome of its depletion^5^,^7^. Premature senescence as a consequence of *BUB1* knockdown has been reported using the same shRNAs employed here^7^, and served as a positive control for senescence induction after lentiviral transduction and W-CIN generation. Cohesin is a multi-protein complex that acts like a molecular adhesive, keeping sister chromatids together until the metaphase to anaphase transition. Once chromosomes are properly aligned and attached to the microtubules, SAC is silenced and the “Anaphase-Promoting Complex” is activated targeting Pituitary tumor transforming 1 (PTTG1/Securin) and Cyclin B1 (CCNB1) for degradation. This process leads to cleavage of cohesin, sister-chromatid separation, and mitotic exit^21^. Mutation or depletion of *SMC1A* in human cells generates W-CIN^5^,^8^, and we demonstrate for the first time, that it induces premature senescence and SASP through expression of *CDKN1A* and *CDKN2A*. Senescence induction through SMC1A deficiency provides a possible explanation for the accelerated aging phenotype presented by patients affected by the cohesinopathy Cornelia de Lange Syndrome (CdLS)^52^. Of note, a novel cohesinopathy caused by a mutation in the cohesin component *SGOL1* shows premature senescence in dermal fibroblasts supporting the link between senescence and cohesin deficiency^53^.

The results obtained upon *BUB1* knockdown were reinforced in *SMC1A*-depleted cells, supporting the hypothesis that W-CIN-induced senescence is likely a general phenomenon. The discrepancy on the levels of W-CIN and the severity of SAFs generated upon depletion of either gene could possibly be explained by variations in the knockdown efficiency. Alternatively, because these genes also have mitosis-independent functions, deregulation of their expression could alter cellular pathways associated with senescence induction, independently of W-CIN. For instance, both BUB1 and SMC1A participate in the DDR^54^,^55^,^56^,^57^. BUB1 also mediates caspase-independent mitotic death in cells prone to chromosome mis-segregation due to low BUB1 expression^58^, which possibly explains slightly higher apoptotic levels in shB2 cells relative to shS2 (Fig. 3g). SMC1A chromosomal distribution is non random and it was found regulating gene expression also in non diving cells^59^. Thus, while our data strongly support W-CIN as an inductor of senescence, we cannot rule out the possibility that depletion of BUB1 or SMC1A promotes irreversible cell cycle arrest per se.

**Figure 8:**
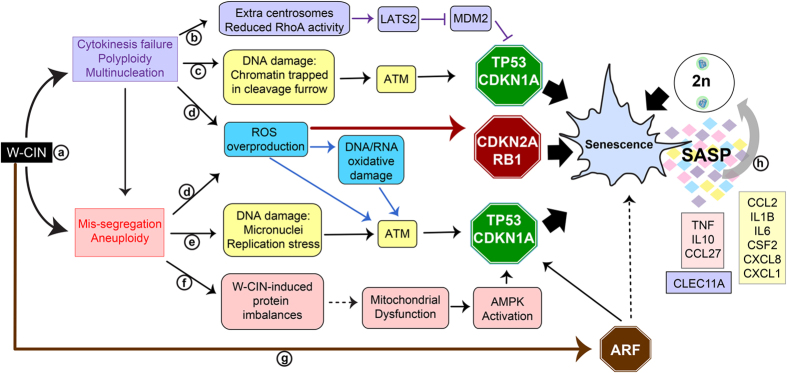
Proposed model for mechanisms of W-CIN induced senescence and SASP. Several mechanisms can explain senescence induction through W-CIN, involving the TP53/CDKN1A, CDKN2A/RB1 or ARF pathways. (**a**) W-CIN generates polyploid and/or multinucleated cells through cytokinesis failure and aneuploid cells through mis-segregation of chromosomes. Because polyploid cells are unstable, they can evolve to an unbalanced aneuploid state. (**b**) Cytokinesis failure leads to extra centrosomes and reduced RhoA activity, which in turn results in indirect stabilization of TP53 via LATS2-inhibition of MDM2 function. (**c**) Chromatin trapped in the cleavage furrow can activate ATM and thus, TP53. (**d**) Polyploid and aneuploid cells generate elevated ROS levels that can activate the CDKN2A/RB1 pathway. Alternatively, ATM activation directly by ROS or via oxidative DNA/RNA damage can also turn on TP53. (**e**) Chromosome mis-segregation potentially produces micronuclei and replication stress, both of which activate DDR, ATM and TP53. (**f**) Aneuploidy-induced protein imbalances caused by gain or loss of specific chromosomes can have 2 consequences: disruption of cellular redox homeostasis leading to ROS overproduction, and mitochondrial dysfunction due to uneven production of mitochondrial proteins. In the latter case, AMPK activation result in TP53-dependent senescence. (**g**) Multinucleation and aneuploidy can up-regulate ARF through DNA damage generation or other pathways, triggering senescence by TP53 activation or trough other TP53 independent mechanisms. (**h**) W-CIN induced SASP comprise factors signature of DNA damage (yellow box), mitochondrial dysfunction (pink box) and by also a novel growth factor CLEC11A (purple box). Factors such as CCL2 and IL1B induce paracrine bystander senescence, potentially spreading the senescence phenotype to normal diploid cells. Nevertheless, all the above-mentioned mechanisms are not mutually exclusive.

Mounting evidence suggests that deviation from diploidy might function similar to DNA damage or OS, as one of the triggers to elicit senescence response^6^,^7^,^16^,^17^. W-CIN, a cellular state with a high propensity for chromosome mis-segregation, generates polyploid and/or multinucleated cells through cytokinesis failure^3^, and these unstable cells can evolve to an unbalanced aneuploid state (Fig. 8)^18^,^19^. We observed no significant differences between the percentages of aneuploid and tetraploid cells (*p* = 0.1491), suggesting that both mis-segregation of chromosomes and cytokinesis failure contributed to ploidy changes. Indeed, W-CIN fibroblasts often had visible chromatin bridges (Fig. 5a), micronuclei (Fig. 5b) and nuclei with distorted or lobated shape (Fig. 5c), suggestive of lagging chromosomes^8^,^51^. Aneuploid cells frequently contained 5, 6 or 7 signals for either chromosome tested (Supplementary Fig. S2b), indicative of tetraploid/octaploid intermediate stages preceding aneuploidy, as previously suggested^18^,^19^.

Several mechanisms have been proposed to explain the cell cycle arrest following changes in ploidy involving the TP53/CDKN1A, CDKN2A/RB1 and/or ARF (p14^ARF^) pathways. Cytokinesis failure leads to extra centrosomes that ultimately reduce RhoA activity in tetraploid cells, which in turn can activate the Large Tumor Suppressor Kinase 2 (LATS2) and the Hippo tumor suppressor pathway. Phosphorylated LATS2 indirectly stabilizes TP53 by inhibiting E3 ubiquitin ligase MDM2^60^. Alternatively, because W-CIN can generate DNA damage^32^,^33^, ATM activation and a persistent DDR could be the signal to trigger senescence through the TP53/CDKN1A pathway. In agreement with this idea, both BUB1 and SMC1A-depleted cultures contained more cells positive for DSBs (γH2AX and 53BP1 foci) than EV, as well as frequent micronuclei and chromatin bridges (Fig. 5a,b). However, the SASP induced by W-CIN may harbor distinguishing features. In fact, some of the W-CIN-induced SASP components overlap with those from DNA damage-induced senescence (CXCL8, CSF2, CCL2, IL6)^48^,^49^, while others are distinct, suggesting that DDR may be only one of the facets of W-CIN-induced senescence. Aneuploid, polyploid and multinucleated cells have increased ROS levels and/or oxidative DNA damage that concurs with TP53 activation^25^,^39^,^61^,^62^, in agreement with our observations of BUB1 and SMC1A-depleted cells containing more cytosolic superoxide and lipofuscin than EV cells. ATM is also activated by ROS, which in turn leads to phosphorylation of TP53 but not of DNA damage-associated proteins like H2AX^63^, suggesting that OS could lead to TP53-mediated arrest in the absence of DSBs. Interestingly, polyploid and multinucleated cells generated by different approaches undergo premature senescence by activation of the CDKN2A/RB1 pathway, with or without DNA damage being detected^62^,^64^,^65^. Because ROS can induce senescence through the CDKN2A/RB1 pathway^61^,^62^, it is tempting to speculate that in polyploid cells, mitotic arrest relies on ROS-induced CDKN2A expression. Moreover, aneuploidy disrupts cellular redox homeostasis^38^, possibly due to imbalances in antioxidant defenses caused by gain or loss of specific chromosomes, resulting in a state of persistent OS^35^. The proteotoxic stress and accumulation of lipofuscin in aneuploids may also underlie other mechanisms causative of OS, such as ROS generation induced by oxidized proteins or iron-bound lipofuscin^29^,^66^. In addition, overproduction of ROS and/or protein imbalances that occur in aneuploid cells, could potentially lead to mitochondrial dysfunction, which has been shown to induce senescence with a distinct SASP comprising by CCL27, TNF and IL10^47^, all of which were significantly more secreted by shS2 cells (TNF also by shB2) (Fig. 6d). Finally, increased ARF levels have also been implicated in senescence induction upon multinucleation and aneuploidy in the presence or absence of DNA damage^6^,^62^ ARF can mediate cell cycle arrest through activation of the TP53 pathway, but also independently of it^67^. Yet, all the above-mentioned mechanisms are not mutually exclusive. Based on our findings and previously published reports, we propose a model in which W-CIN induces senescence though multiple tumor suppressor pathways that converge in the activation of the senescence program and irreversible cell cycle arrest (Fig. 8).

Previous studies suggested that W-CIN is associated with IL6 secretion, which resulted from p53 activation in human cells but was independent of senescence in mice cells^68^,^69^. Multinucleation was also shown to induce senescence and a SASP, with some overlap with the factors found in this study (IL6, CXCL8, IL1B)^62^,^70^. Here, we show that the SASP of W-CIN-induced senescent human cells comprises a variety of cytokines and chemokines previously associated with different senescence conditions^2^,^45^,^46^, but it also induces a novel growth factor CLEC11A, which levels increase with W-CIN severity. The biological functions of W-CIN-induced SASP are diverse: reinforcement of growth arrest in an autocrine feedback loop (CXCL8, CXCL1, IL6)^1^, paracrine induction of senescence in bystander normal cells (CCL2, IL1B)^71^,^72^, promotion of epithelial-mesenchyme transitions and invasiveness (CXCL8, IL6)^48^, stimulation of angiogenesis (CCL2, CSF2, CXCL12)^2^,^73^, tumor immunosuppression (CSF2)^2^, stimulation of pro-inflammatory cytokines, nitric oxide and up-regulation of matrix metalloproteinases (MMPs) (MIF)^74^, inhibition of adipogenesis and keratinocyte differentiation (CCL27, TNF, IL10), among others. The novel factor, CLEC11A, is a glycoprotein secreted by myeloid cells and fibroblasts, which supports the proliferation and differentiation of primitive hematopoietic progenitor cells and acts synergistically with other cytokines including CSF2^75^,^76^. Future studies evaluating the biological effects of this molecule on other cell types are needed to shed light on the relevance of W-CIN-induced senescence. It should be noted that the immunoassay used in this study did not interrogate secreted proteases such as MMPs, extracellular insoluble molecules or TGFB1, all of which are important components of the SASP^2^,^71^. Thus, the SASP presented in our study is likely an underestimation of the potential secretome of W-CIN cells. Moreover, additional variations in SASP composition could be expected depending on the chromosomes that are gained or lost.

In conclusion, our study significantly contributes to the scattered evidence that W-CIN is sufficient to trigger premature senescence and shows that cells deviating from diploidy can affect their microenvironment through the expression of the SASP. The W-CIN-associated secretome has the potential to spread the senescence growth arrest to bystander diploid normal cells, therefore contributing to aging phenotypes and age-related diseases. These findings implicate that aneuploid cells that accumulate during aging in some mammalian tissues may undergo senescence and potentially contribute to inflammation and tissue degeneration though SASP secretion.

## Material and Methods

### Human primary fibroblast (HPF) cultures and growth curve analysis

IMR-90 cells were purchased from ATCC (CCL-186) at PD25 (~passage 12) and cultured in EMEM supplemented with 10% FBS, penicillin and streptomycin in a humidified atmosphere of 5% CO_2_ at 37 °C. The cells were trypsinized and expanded every 3–4 days and their numbers were counted in a hemocytometer to calculate the PDs of each culture. The doubling time (in hours) was calculated with the following formula = h*ln(2)/ln(c2/c1), where c is the number of cells at each time of collection, and ln is a neperian logarithm. SEN fibroblasts were obtained by sub-culturing the same IMR-90 cells until they failed to reach confluency after 2 weeks of culturing (~PD75) and used as positive control for SAFs. Paraquat dichloride hydrate (PQ) induced senescence was triggered as previously described, with minor modifications for human fibroblasts (IMR-90)^50^. Briefly, early passage HPF were exposed to 30 uM of PQ for 10 days and then processed for RNA isolation and SA-β-gal staining. Bleomycin (Bleo) induced senescence was triggered as previously described^31^. Briefly, early passage HPF were exposed to 20 μg/ml Bleo for 2 h with a recovery period of 10 days, after which they were processed for RNA isolation and SA-β-gal staining.

### MEFs isolation and culture

MEFs were isolated from C57Bl/6 mice as follows: torsos from E13.5 embryos were washed and minced in 2 ml of PBS using a syringe and an 18-guage needle. After straining to remove large fragments, the suspension was placed in a 25-cm^2^ flask containing DMEM supplemented with 10% fetal calf serum, penicillin and streptomycin, buffered with bicarbonate and incubated in 10% CO_2_ plus 3% oxygen. After 2 days, cells that grew from tissue fragments were transferred to 10-cm^2^ dishes and cultured to 90% confluency. From this enriched MEF population, 5 × 10^5^ cells were sub-cultured in 10-cm^2^ dishes and considered as passage 1. MEFs were then cultured in atmospheric oxygen (20% - standard culture condition) until they underwent SEN after 8–10 PDs, as previously reported^77^. Senescence induction was validated by observing morphological changes associated with SEN and increased SA-βgal staining (Supplementary Figure S1c,f), as well as failing to detect changes in cell numbers for more than 2 weeks. Two separate cultures (derived from 2 individual embryos) were generated and analyzed.

### shRNA lentiviral transduction

Viral supernatant from the TRC library (The RNAi Consortium – Broad Institute) was produced at the shRNA Core Facility at Albert Einstein College of Medicine using standard protocols^78^. Lentiviral containing either an empty vector (pLKO.1 – no shRNA) or shRNAs targeting *BUB1* or *SMC1A* were used to transfect early passage IMR-90 cells. We first tested which MOI was necessary to achieve a transduction level suitable for our experimental design and found that MOI = 4 resulted in 87% of cells being infected after 48 hours (Supplementary Figure S7) and it was kept consistent throughout the experiments. We initially tested 4 randomly selected shRNAs targeting *BUB1* (shB1–shB4) and other 4 against *SMC1A* (shS1–shS4) and then selected the 2 shRNAs that resulted in greatest gene silencing for each gene (see Supplementary Table S7 for shRNA sequences). In parallel, cells were infected with a lentiviral supernatant containing an empty vector (EV) and used as a control for lentiviral transduction. PD35 fibroblasts (~passage 15) were plated at 4.5 × 10^5^ cells/cm^2^ in 10 cm^2^ plates in culture media containing 1 μg/ml of Polybrene, and lentiviral supernatant was added with a MOI = 4 for 48 h. Infected cells were selected with Puromycin for 10 days after 48 h of lentiviral transduction and then processed for further analysis. Total RNA, DNA and protein were extracted using the AllPrep DNA/RNA/Protein Mini kit (Qiagen) according to the manufacturer’s instruction and quantification performed with Qubit assay kits (dsDNA BR, RNA BR and protein).

### Real-time PCR

Primers for *BUB1, SMC1A, BUB1B, BUB3, CLEC11A*, and *GAPDH* were designed using Primer 3 web tool, choosing amplicons producing 80–100 bp products and excluding amplification of non-specific products by analyzing their sequences against public databases (BLAST). Primers for *SOD1* and *GPX1* were purchased from RealTimePrimers.com. TaqMan probes for *GAPDH* and *CLEC11A* were also designed with Primer 3 web tool, choosing DNA oligos with optimum melting temperature of 60 °C (±1 °C) and GC content of 30%. *GAPDH* probe was conjugated to JOE fluorophore at 5′ and *CLEC11A* with 6-FAM fluorophore, while the 3′ quencher of all probes was ZEN/Iowa BlackFQ. Integrated DNA Technologies (IDT) synthetized both the primers and probes. RNA was reverse-transcribed with Superscript II and random hexamers according to the manufacturer’s instructions. For gene expression analysis we used either the SYBR green detection system or TaqMan probes on an ABI StepOnePlus instrument, according to manufacturer’s cycling conditions. The relative abundances of the mRNAs for the genes of interest were calculated after normalization against *GAPDH* mRNA levels as an internal control. The primers and TaqMan probes sequences used are listed in Supplementary Table S8.

### Western Blotting

Protein lysates (20–35 μg) were separated by electrophoresis in NuPAGE 10% Bis-Tris gels and transferred to PVDF membranes by electro blotting. The membranes were incubated with anti-TUBA4A (Sigma-Aldrich-mouse-T9026) to confirm even loading throughout the samples and anti-SMC1A (Bethyl–rabbit-A300–055A) and anti-BUB1 (Bethyl-rabbit-A300–373A-T) as primary antibodies for the proteins of interest. HRP-conjugated mouse and rabbit IgGs were used as secondary antibodies (Cell Signaling). Target proteins were detected with a chemiluminescent substrate, and films were scanned and converted to grayscale images using Adobe Photoshop CS6.

### IF

Cells plated on coverslips were washed with PBS, fixed with 4% paraformaldehyde, permeabilized with 0.3% Triton X-100 and blocked with 5% goat serum in PBS for 1 h. Cells were incubated with appropriate primary antibodies overnight at 4 °C: anti-MKI67 (eBioscience – 41-5698-80), anti-CDKN1A (Cell signaling – 2947), anti-CDKN2A (Santa Cruz Biotech – sc-56330), anti-phospho-H2AX (Ser139) (Millipore-05–636) and anti-TP53BP1 (Millipore – MAB3802). Cells were then incubated with the appropriate conjugated secondary antibodies (Life technologies) for 1 h: Alexa Fluor 488,555 or 647. Coverslips were then rinsed, dehydrated with ethanol and mounted with ProLong Gold antifade reagent with DAPI for imaging. At least 100 cells were inspected at 40X magnification per condition in 3 independent experiments.

### FISH coupled with IF for BrdU incorporation

To assess DNA replication in human fibroblasts, 10 μM of Bromodeoxyuridine (BrdU) was added to the cells at the 12th day after lentiviral transduction for 24 h. Cells were trypsinized, fixed with ice cold methanol:acetic acid (3:1), dropped into slides and kept in a 37 °C incubator until use. FISH was performed as previously described^9^,^20^, with slight modifications for detection of BrdU and using BAC clones RP11-8L13 (9q21) and RP11-18K11 (9p13) for human chromosome 9 and RP11-51C9 (12p12) and RP11-35G5 (12q14) for human chromosome 12. After overnight hybridization of FISH probes, slides were washed in 0.4X SSC pre-warmed to 74 °C, followed by 4xSSC/0.1%Tween. Slides were processed for IF by standard procedures described above, using a primary anti-BrdU (Sigma-Aldrich – B8434) and a secondary antibody that emits fluorescence in the far-red range (Alexa Fluor 750 nm). Images representing 200 nuclei were randomly acquired and saved as.tiff composite files for all samples from 3 independent experiments. Images were visually inspected and FISH signals manually counted for all cell lines blindly. Ploidy analysis in MEFs (Fig. 1b) was performed as previously described^9^, using the same BAC clones for mouse chromosome 1 and 18.

### Fluorescent image acquisition

Images were acquired with a manual inverted fluorescence microscope (Axiovert 200, Zeiss) with fine focusing oil immersion lens (×40, NA 1.3). The resulting fluorescence emissions were collected using 350-to-470 nm (for DAPI), 436-to-480 nm (for DY-415-dUTP), 470-to-540 nm (for DY-495-dUTP and Alexa Fluor 488), 546-to-600 nm (for DY-590-dUTP an Alexa Fluor 555) and 620-to-700 nm (for DY-647P1-dUTP and Alexa Fluor 647) filters. The microscope was equipped with a Camera Hall 100 and the Applied Spectral Imaging software.

### Analysis of apoptotic bodies

Nuclear morphology was identified using histochemical labeling with DAPI. The same slides used for FISH analysis were inspected for nuclei with fragmented appearance, suggestive of apoptosis. At least 300 cells per conditions were analyzed using 350-to-470 nm filters for DAPI staining at 40X magnification in 3 independent experiments.

### SA-βgal staining

This staining was performed as previously described^28^. Briefly, cells plated on coverslips were washed with PBS and fixed for 5 minutes with 2% formaldehyde/0.2% glutaraldehyde in PBS. Cells were incubated with freshly prepared β-gal staining solution for 24 hours at 37 °C in absence of CO_2_, washed with PBS, counterstained with eosin, rinsed with distilled water and air dried. Coverslips were mounted with permount and analyzed at 10X and 20X magnification using a manual inverted fluorescent microscope equipped with an Axiocam MRm (Zeiss) using the Axiovision 4.7 software. At least 100 cells were inspected per condition in 3 independent experiments.

### Analysis of senescent-like cells and AF by FACS

Increased size and AF (senescent-like phenotype) were measured by FACS according to a published protocol^30^. Briefly, the AF of unfixed cells was measured in the FITC channel and the size was monitored by FSC, after defining the population of live cells in a FSC/SSC dotplot. Both, the percentage of senescent-like cells and the mean intensity of the FITC channel (AF) were quantified for each sample in 3 independent experiments.

### Telomere Length Measurements by Quantitative PCR

Genomic DNA samples at a minimum concentration of 20 ng/μL were processed by the Rabinovitch Lab (Department of Pathology, University of Washington) using a protocol previously described with minor modifications^34^. Briefly, two PCRs were performed: the first one to amplify the telomeric DNA and the second one to amplify a single-copy control gene (ribosomal protein lateral stalk subunit P0 – RPLP0). This provides an internal control to normalize the starting amount of DNA. A four-point standard curve (2-fold serial dilutions from 5 to 0.625 ng of DNA) was included in all PCRs to allow the transformation of cycle threshold (Ct) into nanograms of DNA. Samples were run in triplicate, and the median was used for calculations. The amount of telomeric DNA (T) was divided by the amount of single-copy control gene DNA (S), producing a relative, unit-less measurement of telomere length (T/S ratio). Two control samples were run in each experiment to allow for normalization between experiments, and periodic reproducibility experiments were performed. Inter -assay coefficient of variation (CV) was 0.06. The values obtained for each cell line were normalized against control EV cells in 2 independent experiments.

### Evaluation of ROS (cytosolic superoxide) production by FACS

After 13 days of lentiviral transduction, cells were incubated with 5 μM of the superoxide indicator Dihydroethidium (DHE) as previously described^40^, and average fluorescence was determined for each sample in 3 independent experiments.

### FACS data analysis

Results recorded for senescent-like phenotypes, ROS production and AF were analyzed with the single cell analysis software FlowJo v10.1.

### Analysis of DNA and RNA oxidation

The amount of oxidative base damage in both DNA and RNA was evaluated by using the EIA kit (Enzyme Immunoassay, Cayman chemical) following manufacturer’s instructions. The level of damage was expressed in picograms of guanine oxidation per milliliter (pg/ml) of either DNA or RNA isolated from cell lines and plotted as oxidative damage relative to EV cells.

### SASP analysis

Conditioned medium was generated by adding fresh EMEM without FBS for 24 h to cells. For the preliminary SASP screen, the concentration of 48 factors in the culture medium of EV or shS2 cells were determined using the Bio-Plex Pro Human Cytokine 27-plex and 21-plex immunoassay kits (Bio-Rad) according to the manufacturer’s protocols. The 48 cytokines interrogated were: CXCL8, CXCL10, CCL2, IL1, TNF, CXCL1, CSF2, IL6, CCL27, CXCL12, IL15, IL12B, MIF, NGF, CLEC11A, IL10, KITLG, IL2RA, CSF1, IL18, IL1A, VEGFA, IL16, HGF, IL9, IFNA2, CXCL9, CCL11, CCL3, CCL4, CCL5, CCL7, CSF3, FGF2, IFNG, IL12B, IL13, IL17A, IL1RN, IL2, IL3, IL4, IL5, IL7, LIF, LTA, PDGFB, TNFSF10. For the validation of SASP factors of interest, a human ProcartaPlex custom-designed immunoassay kit (Affymetrix eBioscience) was used according to the manufacturer’s protocols. We selected 13 SASP components based on significant *p* value or high fold differences between EV and shS2: CCL2, CCL27, CLEC11A, CSF2, CXCL1, CXCL10, CXCL12, CXCL8, IL10, IL1B, IL6, MIF and TNF. Six biological replicates were analyzed for all samples (EV, shB1, shB2, shS1, shS2), except for SEN (n = 5). The concentrations of each factor were calculated according to the standard curves, which were generated by the standard mixtures provided with each assay kit. Biological replicates with fluorescent levels below the detection range were excluded from the analyses. For factors expressed below the detection level, we assigned a nominal value of 0.01 to prevent zero values in the statistical analysis. Values above the detection range (CXCL8 only) was assigned as the double of the highest value detected for this cytokine, in order to provide an estimated quantification for SEN cells and to enable statistical evaluation. The observed concentrations obtained with this assay were divided by the number of cells previously present in each sample for normalization of the data.

### Statistical analysis

All the statistical analyses were performed using the software GraphPad Prism 6.0 and significance was calculated with 95% confidence interval (alpha <0.05). All the analysis were carried on using One-Way ANOVA comparing the average of each sample to the average of EV control cells without correcting for multiple comparisons, unless otherwise indicated in the text or figure legends. Correlation analyses were performed by computing the percentages of not 2n cells with the expression of the various parameters of interest analyzed in this study (i.e. MKI67, CDKN1A and CDKN2A expression, BrdU incorporation, apoptotic bodies, SA-βgal staining, DNA damage, ROS production and levels of SASP components), to determine the Pearson correlation coefficient (*r*) and the *p* value in each case. Data used for correlation plots are presented in the Supplementary material (Supplementary Tables S3 and S6).

## Additional Information

**How to cite this article**: Andriani, G. A. *et al*. Whole Chromosome Instability induces senescence and promotes SASP. *Sci. Rep*. **6**, 35218; doi: 10.1038/srep35218 (2016).

## Supplementary Material

Supplementary Information
