# Supplementary material for: Whole Chromosome Instability induces senescence and promotes SASP
Source: Sci Rep. 2016 Oct 12;6:35218. doi: 10.1038/srep35218 (PMC5059742; doi:10.1038/srep35218)

Whole Chromosome Instability induces senescence and promotes SASP

Grasiella Angelina Andriani, Vinnycius Pereira Almeida, Francesca Faggioli, Maurizio Mauro, Wanxia Li Tsai, Laura Santambrogio, Alexander Maslov, Massimo Gadina, Judith Campisi, Jan Vijg and Cristina Montagna.

Supplementary information

Supplementary Tables:

Table S1. Distribution of ploidy changes observed during replicative senescence and total number of cells analyzed for each cell line.

	Total %Aneuploid	%4n	%3n	%1n	%>5n	%2n	%Non-diploid	Cells counted
**HPF PD42**	4.0	4.5	0.5	0.2	0.7	90.1	9.9	554
**HPF PD56**	10.0	7.5	1.1	0.4	1.4	79.6	20.4	279
**HPF PD74**	22.9	26.6	1.5	0.0	11.1	37.8	62.2	458
**MEF PD04-1**	5.1	30.3	0.0	0.0	0.0	64.6	35.4	99
**MEF PD04-2**	2.0	42.6	0.0	0.0	1.0	54.5	45.5	101
**MEF PD10-1**	19.6	36.1	2.1	0.0	9.3	33.0	67.0	97
**MEF-PD10-2**	16.1	36.6	0.0	0.0	6.5	40.9	59.1	93

Table S2. Distribution of ploidy changes detected in each cell line per biological replicate (1, 2 and 3), and frequency BrdU positive cells as detected by immunofluorescence.

	%Aneuploid	%4n	%3n	%1n	%>5n	%2n	%Non-diploid	% BrdU
**EV1**	1.9	2.9	0.5	0.0	0.5	94.2	5.8	83.1
**EV2**	0.0	3.0	0.0	0.0	0.0	97.0	3.0	82.1
**EV3**	5.0	5.0	0.0	0.0	1.0	88.9	11.1	72.4
**shB1-1**	6.3	8.9	0.0	1.6	1.0	82.3	17.7	72.9
**shB1-2**	1.5	10.4	0.0	0.0	0.0	88.1	11.9	72.3
**shB1-3**	18.1	4.5	1.0	0.0	0.5	75.9	24.1	81.9
**shB2-1**	6.5	9.7	0.0	0.0	0.0	83.9	16.1	61.3
**shB2-2**	12.4	9.4	0.5	0.0	3.0	74.8	25.2	64.4
**shB2-3**	3.6	14.3	0.5	0.0	2.0	79.6	20.4	68.4
**shS1-1**	18.6	11.3	2.0	0.5	1.5	66.2	33.8	51.0
**shS1-2**	17.9	18.4	2.0	0.0	3.5	58.2	41.8	52.5
**shS1-3**	4.0	17.4	0.5	0.0	1.0	77.1	22.9	49.8
**shS2-1**	22.3	11.4	2.6	0.5	3.1	60.1	39.9	37.3
**shS2-1**	20.9	18.4	2.5	0.0	2.5	55.7	44.3	38.5
**shS2-3**	10.0	23.0	1.0	0.0	1.0	65.0	35.0	39.0
**SEN1**	9.2	19.5	0.5	0.0	1.5	69.2	30.8	19.8
**SEN2**	10.1	23.1	0.5	0.5	1.9	63.9	36.1	21.6

Table S3. Summary data computed for correlation plots. Pearson correlation coefficient (r) and p values for each correlation analysis are presented. The percentage of not 2n cells in each replicate sample (X axis) was plotted against the different parameters analyzed (Y axis). The main text includes plots depicting EV, shB2, shS2 and SEN cultures. All correlations were statically significant (p<0.05), except for "%Not 2n cells vs. % Apoptotic bodies".

	X axis	Y axis									
**Sample**	**%Not 2n cells**	**%MKI67 +**	**%BrdU +**	**%Apoptotic bodies**	**%βgal +**	**%SEN-like**	**%CDKN2A +**	**%CDKN1A +**	**%H2AX +**	**DHE intensity**	**%53BP1 +**
**EV-1**	5.8	63.1	83.1	1.5	11.7	10.7	18.6	31.8	22.7	4722	62.3
**EV-2**	3.0	69.8	82.1	1.6	8.1	18.1	17.6	25.0	20.8	6555	33.3
**EV-3**	11.1	62.8	72.4	1.3	8.8	14.5	17.1	40.2	27.5	4870	34.5
**shB2 -1**	16.1	34.4	61.3	2.8	57.7	36.5	37.6	46.5	32.0	5920	63.3
**shB2 -2**	25.3	28.6	64.4	4.1	47.5	34.8	41.5	50.9	38.1	5988	57.3
**shB2 -3**	20.4	34.1	67.9	3.3	61.1	36.3	33.0	55.3	30.4	6335	63.4
**shS2-1**	39.9	20.0	37.3	3.7	-	57.6	66.2	67.5	43.8	11400	71.1
**shS2-2**	44.3	20.0	38.5	2.4	77.8	61.7	68.7	61.6	46.8	12500	77.8
**shS2-3**	35.0	12.7	39.0	1.2	72.2	57.4	62.7	63.9	48.6	10800	64.3
**SEN-1**	-	14.7	-	3.9	76.3	54.1	59.6	77.5	72.0	8238	79.7
**SEN-2**	30.8	10.3	19.8	3.6	82.9	53.9	49.5	79.4	69.7	8214	92.3
**SEN-3**	36.1	9.5	21.6	1.3	81.4	36.8	54.2	77.8	84.7	4664	83.8
**Pearson correlation coefficient (r)**		**-0.907**	**-0.8682**	**0.2884**	**0.8989**	**0.9116**	**0.974**	**0.8711**	**0.7137**	**0.72**	**0.7347**
***p* value (two-tailed)**		**0.0001**	**0.0005**	**0.3897**	**0.0004**	**< 0.0001**	**< 0.0001**	**0.0005**	**0.0136**	**0.0125**	**0.01**
**Significant? alpha<0.05**		**Yes**	**Yes**	**No**	**Yes**	**Yes**	**Yes**	**Yes**	**Yes**	**Yes**	**Yes**

Table S4. Values of the SASP factors secreted 2 fold or more by shS2 cells relative to EV detected in the preliminary SASP screen. For each sample, the average and standard deviation (s.d.) concentration values for each component (pg/ml) are shown. The results are listed in order of fold difference expression, indicating the *p* values for each SASP component (generated from a t-test analysis between EV and shS2 values – n=2). Official gene symbol is followed by previous gene symbol and spelled full names within brackets.

Gene	EV Average (pg/ml)	EV (s.d.)	shS2 Average (pg/ml)	shS2 (s.d.)	Fold difference	Significant? (alpha<0.05)	*p* value (two-tailed)
CXCL8/IL-8 (C-X-C motif chemokine ligand 8)	7.0	9.8	1917.9	1170.9	273.3	No	0.147
CXCL10/IP-10 (C-X-C motif chemokine ligand 10)	0	0	96.0	55.6	96.0	No	0.135
CCL2/MCP-1 (C-C motif chemokine ligand 2)	4.9	6.9	156.6	26.6	32.2	Yes	0.016
IL1B/IL-1β (Interleukin 1 beta)	0.1	0	0.9	0.2	16.7	Yes	0.030
TNF/TNF-α (Tumor necrosis factor)	0.6	0.1	8.8	0.8	14.8	Yes	0.005
CXCL1/GROα (C-X-C motif chemokine ligand 1)	10.1	14.3	121.9	172.5	12.1	No	0.457
CSF2/GM-CSF (Colony stimulating factor 2)	2.1	3.0	20.2	4.4	9.6	Yes	0.041
IL6/IL-6 (Interleukin 6)	2.5	3.1	23.1	10.3	9.4	No	0.114
CCL27/CTACK (C-C motif chemokine ligand 27)	0.9	1.3	7.1	4.6	7.8	No	0.208
CXCL12/SDF-1a (C-X-C motif chemokine ligand 12)	12.2	17.3	68.2	11.3	5.6	No	0.062
IL15/IL-15 (Interleukin 15)	0.3	0	1.1	0.8	4.0	No	0.293
IL12B (Interleukin 12B)	4.1	5.5	13.7	2.4	3.3	No	0.152
MIF (Macrophage migration inhibitory factor)	89.2	11.8	292.1	228.1	3.3	No	0.336

NGF/b-NGF (Nerve growth factor)	1.4	0.2	4.5	1.6	3.2	No	0.113
CLEC11A/SCGF-b (C-type lectin domain family 11 member A)	135.8	91.1	422.0	147.6	3.1	No	0.145
IL10/IL-10 (Interleukin 10)	1.1	1.6	3.0	4.2	2.6	No	0.611
KITLG/SCF (KIT ligand)	1.1	0.9	2.5	0.1	2.2	No	0.160

Table S5. Validation of W-CIN-induced SASP components. The secreted factors that were tested are listed in alphabetical order and indicated as the concentration (pg/ml) measured in the conditioned medium of EV, Bub1-, SMC1A-depleted and SEN cells, respectively. For each sample, the average and standard deviation (s.d.) concentration values for each component are shown. The fold differences relative to control EV cells, as well as the *p* value generated from One-way ANOVA analysis comparing all samples to EV are also shown.

		Gene	EV Average (pg/ml)	EV (s.d.)	shB1 Average (pg/ml)	shB1 (s.d.)	Fold difference relative to EV	Significant? (alpha<0.05)	*p* value (two-tailed)
BUB1-depleted	shB1	*CCL2 (MCP-1)*	4.402	1.471	37.002	17.269	8.4	No	0.4819
		CCL27 (CTACK)	0.467	0.121	1.632	0.527	3.5	No	0.3736
		CLEC11A (SCGF-b)	376.129	45.857	835.251	162.340	2.2	No	0.5198
		CSF2 (GM-CSF)	0.631	0.212	0.901	0.061	1.4	No	0.763
		CXCL1 (GROα)	1.215	0.645	0.531	0.132	0.4	No	0.8221
		CXCL10 (IP-10)	0.049	0.012	0.231	0.291	4.8	No	0.8554
		CXCL12 (SDF-1a)	4.014	0.960	6.037	3.008	1.5	No	0.5837
		CXCL8 (IL8)	0.744	0.236	5.215	3.821	7.0	No	0.9427
		IL10	0.074	0.080	0.173	0.074	2.3	No	0.841
		IL1B	0.001	0.000	0.002	0.001	1.4	No	0.994
		IL6	2.734	1.551	23.598	16.623	8.6	No	0.1891
		MIF	3.055	0.854	9.938	3.504	3.3	No	0.4388
		TNF	0.053	0.021	0.161	0.046	3.1	No	0.2379
		**Gene**	**EV Average (pg/ml)**	**EV (s.d.)**	**shB2 Average (pg/ml)**	**shB2 (s.d.)**	**Fold difference relative to EV**	**Significant? (alpha<0.05)**	***p* value (two-tailed)**
	shB2	*CCL2 (MCP-1)*	4.402	1.471	35.866	15.600	8.1	No	0.497
		CCL27 (CTACK)	0.467	0.121	2.565	1.973	5.5	No	0.2086
		CLEC11A (SCGF-b)	376.129	45.857	1817.459	570.667	4.8	No	0.0786
		CSF2 (GM-CSF)	0.631	0.212	0.937	0.921	1.5	No	0.6293

		CXCL1 (GROα)	1.215	0.645	0.920	0.786	0.8	No	0.9155
		CXCL10 (IP-10)	0.049	0.012	0.193	0.235	4.0	No	0.8821
		CXCL12 (SDF-1a)	4.014	0.960	4.672	4.409	1.2	No	0.8523
		CXCL8 (IL8)	0.744	0.236	3.320	2.300	4.5	No	0.9761
		IL10	0.074	0.080	0.531	0.720	7.2	No	0.4666
		IL1B	0.001	0.000	0.001	0.001	1.1	No	0.9989
		IL6	2.734	1.551	9.155	5.749	3.3	No	0.693
		MIF	3.055	0.854	11.115	1.574	3.6	No	0.4058
		TNF	0.053	0.021	0.319	0.149	6.0	Yes	0.0201

SMC1A -depleted

shS1

Gene	EV Average (pg/ml)	EV (s.d.)	shS1 Average (pg/ml)	shS1 (s.d.)	Fold difference relative to EV	Significant? (alpha<0.05)	*p* value (two-tailed)
CCL2 (MCP-1)	4.402	1.471	86.744	54.639	19.7	No	0.1159
CCL27 (CTACK)	0.467	0.121	2850.731	1827.076	6108.9	No	0.308
CLEC11A (SCGF-b)	376.129	45.857	2.143	3.027	0.0	Yes	0.0132
CSF2 (GM-CSF)	0.631	0.212	0.038	0.050	0.1	No	0.5099
CXCL1 (GROα)	1.215	0.645	1.235	1.755	1.0	No	0.9944
CXCL10 (IP-10)	0.049	0.012	3.903	4.450	80.2	Yes	0.0079
CXCL12 (SDF-1a)	4.014	0.960	9.450	12.376	2.4	No	0.2625
CXCL8 (IL8)	0.744	0.236	24.331	30.952	32.7	No	0.7324
IL10	0.074	0.080	0.219	0.374	3.0	No	0.7939
IL1B	0.001	0.000	0.002	0.002	1.5	No	0.993
IL6	2.734	1.551	18.527	15.946	6.8	No	0.3743
MIF	3.055	0.854	23.326	7.588	7.6	Yes	0.0499
TNF	0.053	0.021	0.089	0.015	1.7	No	0.7494

shS2

Gene	EV Average (pg/ml)	EV (s.d.)	shS2 Average (pg/ml)	shS2 (s.d.)	Fold difference relative to EV	Significant? (alpha<0.05)	*p* value (two-tailed)
CCL2 (MCP-1)	4.402	1.471	245.152	166.934	55.7	Yes	0.0002
CCL27 (CTACK)	0.467	0.121	5.090	4.568	10.9	Yes	0.0142
CLEC11A (SCGF-b)	376.129	45.857	3092.486	1422.508	8.2	Yes	0.0042
CSF2 (GM-CSF)	0.631	0.212	3.202	2.416	5.1	Yes	0.0044
CXCL1 (GROα)	1.215	0.645	17.205	12.003	14.2	Yes	0.0001
CXCL10 (IP-10)	0.049	0.012	6.090	1.767	125.1	Yes	0.0003
CXCL12 (SDF-1a)	4.014	0.960	54.133	10.738	13.5	Yes	< 0.0001
CXCL8 (IL8)	0.744	0.236	310.829	273.745	418.0	Yes	0.0008

		IL10	0.074	0.080	1.470	1.881	19.9	Yes	0.0432
		IL1B	0.001	0.000	0.699	0.593	523.0	Yes	< 0.0001
		IL6	2.734	1.551	63.525	51.904	23.2	Yes	0.0033
		MIF	3.055	0.854	60.870	31.504	19.9	Yes	< 0.0001
		TNF	0.053	0.021	0.484	0.272	9.2	Yes	0.001

	Gene	EV Average (pg/ml)	EV (s.d.)	SEN Average (pg/ml)	SEN (s.d.)	Fold difference relative to EV	Significant? (alpha<0.05)	*p* value (two-tailed)
**SEN**	*CCL2 (MCP-1)*	4.402	1.471	277.703	192.630	63.090	Yes	0.0009
	CCL27 (CTACK)	0.467	0.121	2.315	1.160	4.961	No	0.1349
	CLEC11A (SCGF-b)	376.129	45.857	1443.024	300.854	3.837	No	0.0815
	CSF2 (GM-CSF)	0.631	0.212	6.406	1.129	10.148	Yes	< 0.0001
	CXCL1 (GROα)	1.215	0.645	125.527	90.238	103.336	Yes	0.0001
	CXCL10 (IP-10)	0.049	0.012	26.977	15.297	554.233	Yes	< 0.0001
	CXCL12 (SDF-1a)	4.014	0.960	64.369	38.901	16.035	Yes	0.0002
	CXCL8 (IL8)	0.744	0.236	386.169	164.381	519.378	Yes	< 0.0001
	IL10	0.074	0.080	0.482	0.242	6.519	No	0.3463
	IL1B	0.001	0.000	5.190	2.873	3882.364	Yes	< 0.0001
	IL6	2.734	1.551	1643.687	746.793	601.275	Yes	< 0.0001
	MIF	3.055	0.854	24.642	8.639	8.065	Yes	0.0111
	TNF	0.053	0.021	0.743	0.448	14.106	Yes	0.0008

Table S6. Correlation analysis between the percentages of not 2n cells and secretion levels of individual SASP components (CLEC11A, CCL2, CCL27 and MIF) including the Pearson correlation coefficient (r) and *p* values that were found statistically significant. The average percentage of not 2n cells in each sample (X axis) is plotted against the average levels of each factor analyzed (Y axis).

	**X axis**		**Y axis**							
**Sample**	**% Average Not 2n cells**	**% Not 2n cells (s.d.)**	**Average CLEC11A (pg/ml)**	**CLEC11A (s.d.)**	**Average CCL2 (pg/ml)**	**CCL2 (s.d.)**	**Average CCL27 (pg/ml)**	**CCL27 (s.d.)**	**Average MIF (pg/ml)**	**MIF (s.d.)**
**EV**	6.6	4.1	376.1	45.9	4.4	1.5	0.5	0.1	3.1	0.9
**shB1**	17.9	6.1	835.3	162.3	37.0	17.3	1.6	0.5	9.9	3.5
**shB2**	20.6	4.6	1817.5	570.7	35.9	15.6	2.6	2.0	11.1	1.6
**shS1**	32.8	9.5	2850.7	1827.1	86.7	54.6	2.1	3.0	23.3	7.6
**shS2**	39.7	4.6	3092.5	1422.5	245.2	166.9	5.1	4.6	60.9	31.5
**SEN**	33.4	3.7	1443.0	300.9	277.7	192.6	2.3	1.2	24.6	8.6
**Pearson correlation coefficient (*r*)**			**0.8619**		**0.8318**		**0.8221**		**0.8534**	
***p* value (two-tailed)**			**0.0273**		**0.0401**		**0.0447**		**0.0306**	
**Significant? alpha<0.05**			**Yes**		**Yes**		**Yes**		**Yes**	

Table S7. shRNA sequences of the targets used in this study.

Target gene	shRNA ID	Sense sequence 5' - 3'
BUB1	shB1	CCTGGGTCAGAGTATAGATAT
BUB2	shB2	TACAACAGTGACCTCCATCAA
BUB3	shB3	CGAGGTTAATCCAGCACGTAT
BUB4	shB4	AGAAATACAATCAACGGAGAA
SMC1A	shS1	GCCGGGACTGTATTCAGTATA
SMC1A	shS2	CCAACATTGATGAGATCTATA
SMC1A	shS3	CCAACAAGGAAATGACCCATT
SMC1A	shS4	CGACAGATTATCGGACCATTT

Table S8. Sequences of the human primers used for qRT-PCR.

Gene	Fwd primer 5'-3'	Rev primer 5'-3'	TaqMan probe
**BUB1**	GGTGGTGCCTTCAAGGGATGG	GCAGCAGGCTGGCTCAGACG	SYBR green detection
**SMC1A**	AGGCCCTCATTGAGATTGAC	GCAATACGCTGAAGCACACT	SYBR green detection
**BUB3**	CGCATGCCTGGAGTGGAGGA	GGGGCATCATGGGTCCCAAC	SYBR green detection
**BUB1B**	CGTGGCAATACAGCTTCACT	AGGCTTTCTGGTGCTTAGGA	SYBR green detection
**SOD1**	TCAGGAGACCATTGCATCAT	GAATGTTTATTGGGCGATCC	SYBR green detection
**GPX1**	TTGACATCGAGCCTGACATC	ACTGGGATCAACAGGACCAG	SYBR green detection
**GAPDH**	TCAAGAAGGTGGTGAAGCAG	CGCTGTTGAAGTCAGAGGAG	CCTCAAGGGCATCCTGGGCT
**CLEC11A**	GGCTAGTGACCTGCACACAG	GCACCCAAAGTTCCAGATGT	CTCGGCAGTTCCCAGAGGCC

Supplementary figures

Fig S1. Primary mammalian cells *in vitro* show accumulation of ploidy changes as they approach SEN. (a, b, d and e) Representative images of four-color interphase-FISH in HPFs (**a** and **b**) and MEFs (**d** and **e**) showing diploid (**a** and **d**), tetraploid (**b**) and aneuploid nuclei (**e**). (**c and f**) Bright field representative images of senescent HPF (**c**) and MEFs (**f**) stained for SA-βgal.

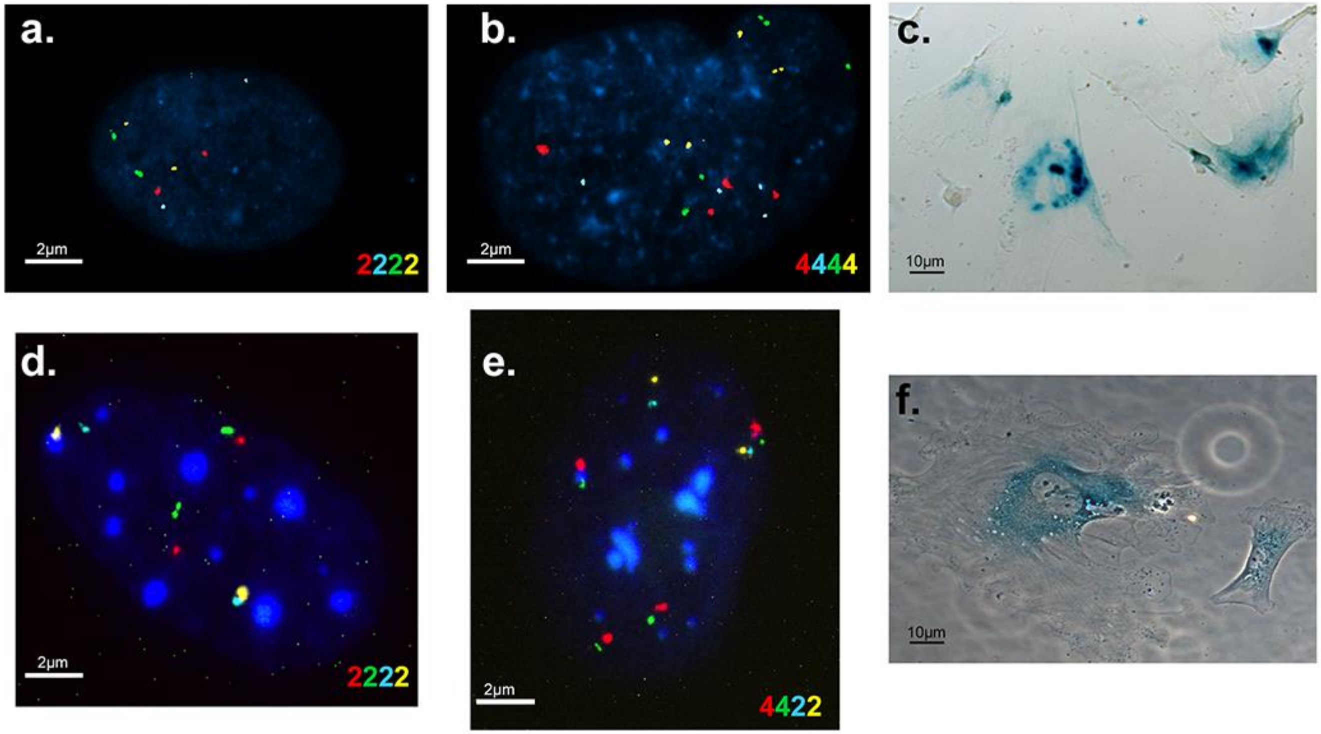

Fig S2. Depletion of *BUB1* or *SMC1A* results in W-CIN, reduction of DNA replication and negligible apoptosis in human fibroblasts. (a-c) Representative interphase FISH images showing the hybridization signals that were detected. The numbers of signals for each chromosome are indicated with the correspondent color of fluorophore used for staining. **(a)** Diploid nuclei (2222). **(b)** Nuclei containing 6 copies of each chromosome (6666), and morphology suggestive of abnormal mitosis. **(c)** Tetraploid nuclei (4444). Representative images of BrdU positive **(d),** and negative **(e)** nuclei containing 2 hybridization signals for chromosome 9 in the Cy5 channel. **(f)** Representative image of nuclei with fragmented appearance, suggestive of apoptotic bodies.

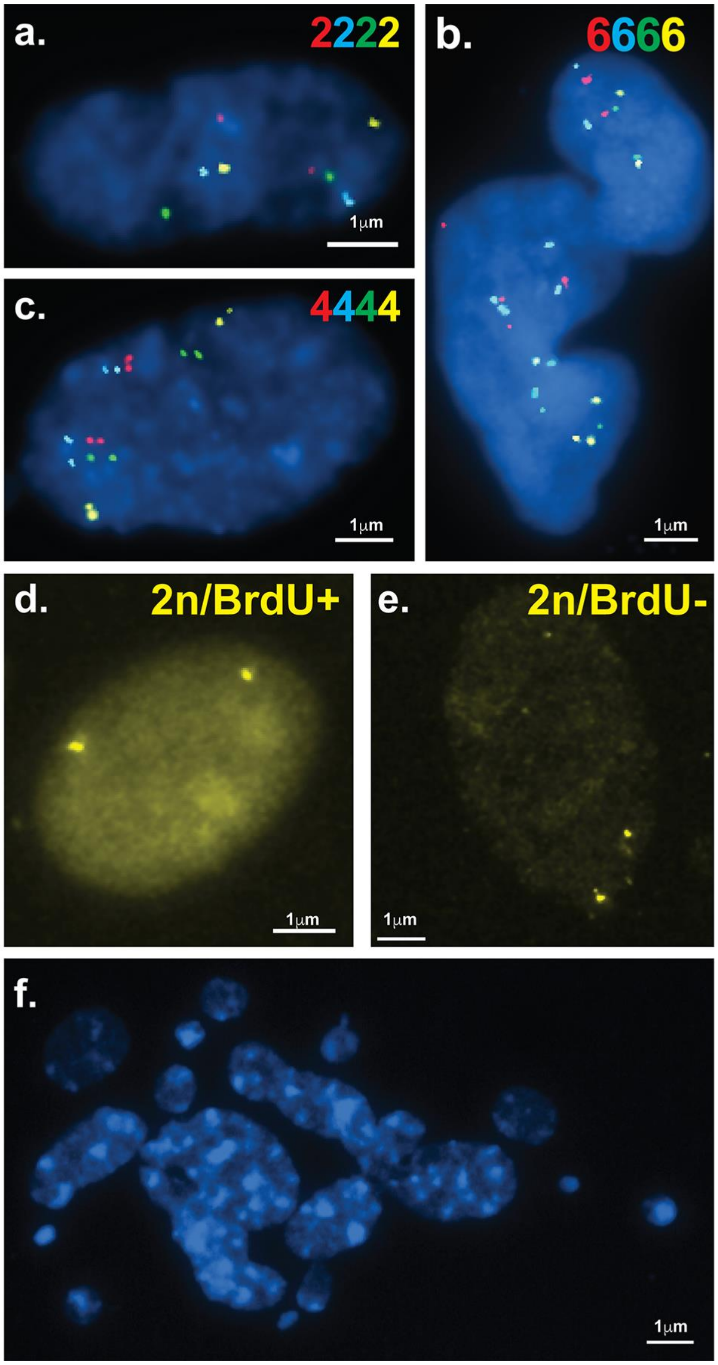

Fig S3. Down-regulation of BUB1 and SMC1A shifts cells towards a senescent-like phenotype. Representative FACS diagrams depicting the senescent-like population (i.e. high size and/or high AF) and respective percentage of these cells in each culture. Forward scatter channel (FSC-A) in X-axis depicts cell size and autofluorescence channel (FITC-A) in Y-axis depicts lipofuscin content. EV=empty vector; SEN= replicative senescence; shB2= BUB1-depleted; shS2= SMC1-depleted.

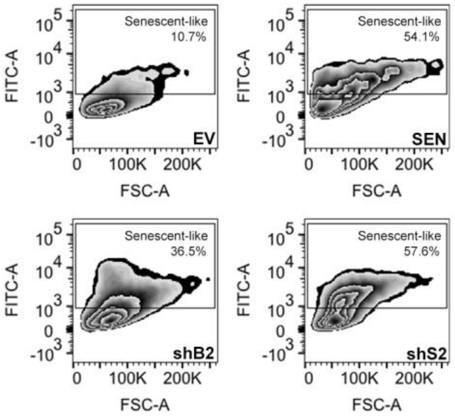

Fig S4. W-CIN-induced senescence is associated with generation of DNA DSBs. (a) Quantification of number of 53BP1 foci per nucleus in each culture. **(b)** Correlation plot between the percentages of not-diploid and 53BP1 foci containing cells. (*) Indicates significant differences (*p*<0.05) from EV cells by One-way ANOVA. Data are mean ±SD (n=3).

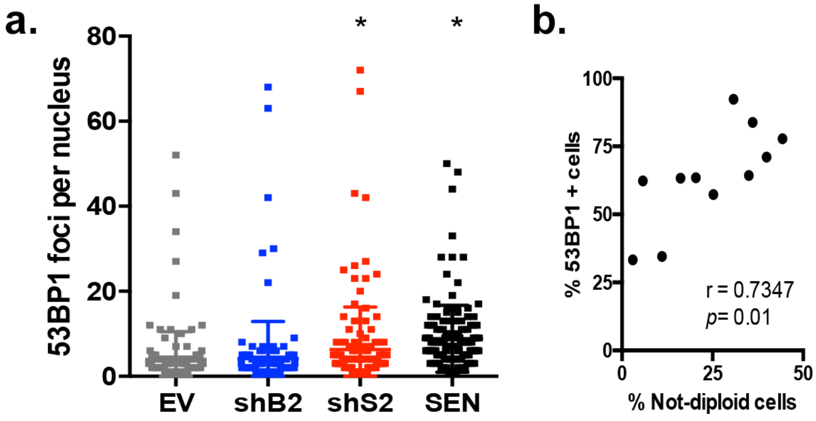

Fig S5. SASP analysis of W-CIN cells. Plots depicting the amount of each of the SASP factors tested (pg/ml) in the CM of all cell lines. (*) Indicates significant differences ($p<0.05$) from EV cells by One-way ANOVA. Data are mean ±SD (n=6, n=5 for SEN).

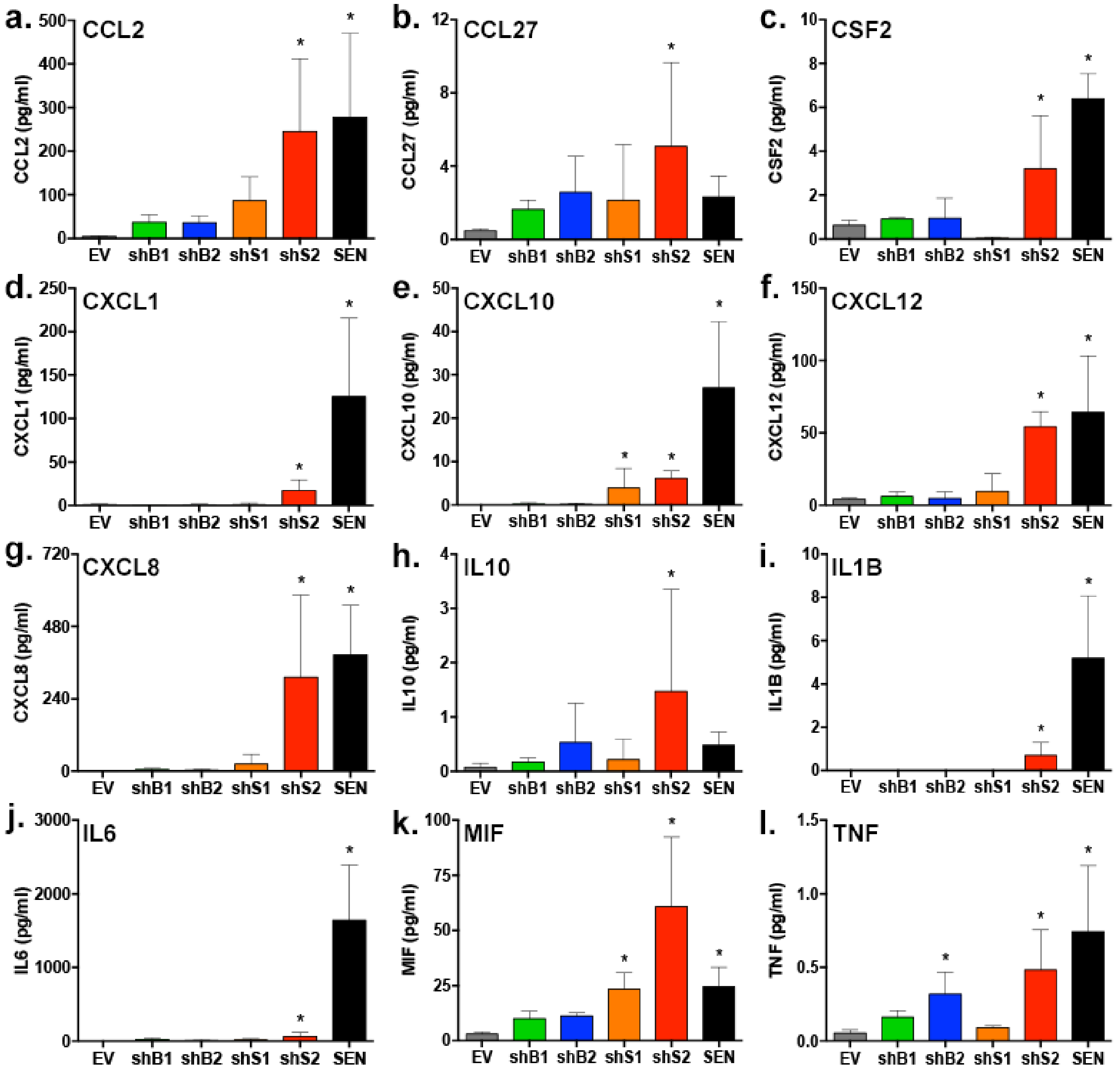

Fig S6. PQ and Bleo-induced senescence do not up-regulate CLEC11A. Bright field representative images of (**a**) PQ- and (**b**) Bleo-induced HPF senescent fibroblasts stained for SA-βgal.

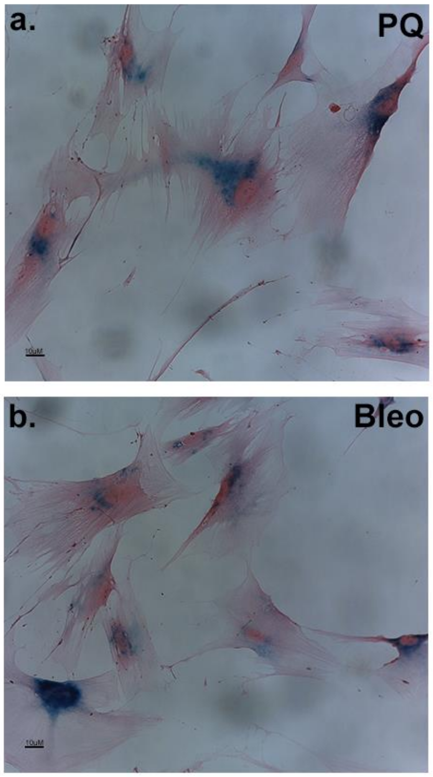

Fig S7. Efficiency of lentiviral transduction. Plot representing the percentage of cells infected by lentivirus at the three multiplicity of infection tested (MOI)(n=2).

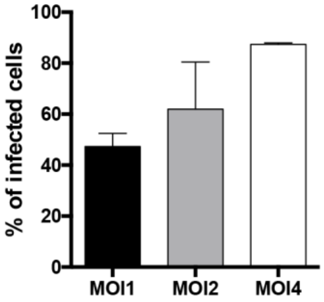

Supplement: Supplementary Information [file srep35218-s1.pdf]
